# Risk factors and 26-years worldwide prevalence of endoscopic erosive esophagitis from 1997 to 2022: a meta-analysis

**DOI:** 10.1038/s41598-023-42636-7

**Published:** 2023-09-14

**Authors:** Andro Pramana Witarto, Bendix Samarta Witarto, Shidi Laras Pramudito, Lintang Cahyaning Ratri, Nabilah Azzah Putri Wairooy, Tiffany Konstantin, Achmad Januar Er Putra, Citrawati Dyah Kencono Wungu, Annisa Zahra Mufida, Arief Gusnanto

**Affiliations:** 1 Medical Program, Faculty of Medicine, Universitas Airlangga, Surabaya, Indonesia; 2 Department of Physiology and Medical Biochemistry, Universitas Airlangga, Jl. Mayjen Prof. Dr. Moestopo No. 47, Surabaya, 60132 Indonesia; 3 Institute of Tropical Disease, Universitas Airlangga, Surabaya, Indonesia; 4 Department of Internal Medicine, Dr. Soetomo General Hospital, Faculty of Medicine, Universitas Airlangga, Surabaya, Indonesia; 5 Department of Internal Medicine, Universitas Airlangga Hospital, Faculty of Medicine, Universitas Airlangga, Surabaya, Indonesia; 6 School of Mathematics, University of Leeds, Leeds, UK

**Keywords:** Gastrointestinal diseases, Diseases, Gastroenterology, Gastrointestinal diseases, Gastrointestinal system, Oesophagogastroscopy, Medical research, Epidemiology, Outcomes research, Risk factors, Signs and symptoms, Digestive signs and symptoms

## Abstract

Erosive esophagitis (EE) is the part of gastroesophageal reflux disease (GERD) spectrum and may progress to esophageal adenocarcinoma. Due to its progressivity and unclear prevalence, we aim to identify the factors contributing in EE to decide the need for further examination. We performed a PRISMA 2020-based systematic search through PubMed and other resources up to June 2, 2022. Study quality was assessed using the Newcastle–Ottawa Scale (NOS). The odds ratio (OR) of each factor and worldwide prevalence of EE were measured. There are 114 observational studies included with a total of 759,100 participants. Out of 29 factors, the significant risk factors are age ≥ 60 y.o. (OR 2.03 [1.81–2.28]), White/Caucasian (OR 1.67 [1.40–1.99]), unmarried (OR 1.08 [1.03–1.14]), having GERD ≥ 5 years (OR 1.27 [1.14–1.42]), general obesity (OR 1.78 [1.61–1.98]), central obesity (OR 1.29 [1.18–1.42]), diabetes mellitus (DM) (OR 1.24 [1.17–1.32]), hypertension (OR 1.16 [1.09–1.23]), dyslipidemia (OR 1.15 [1.06–1.24]), hypertriglyceridemia (OR 1.42 [1.29–1.57]), hiatal hernia (HH) (OR 4.07 [3.21–5.17]), and non-alcoholic fatty liver disease (NAFLD) (OR 1.26 [1.18–1.34]). However, *H. pylori* infection (OR 0.56 [0.48–0.66]) and atrophic gastritis (OR 0.51 [0.31–0.86]) are protective towards EE. This study demonstrates that age, ethnicity, unmarried, long-term GERD, metabolic diseases, HH, and NAFLD act as risk factors for EE, whereas *H. pylori* infection and atrophic gastritis act as protective factors. These findings may enable a better understanding of EE and increase greater awareness to address its growing burden.

## Introduction

Gastroesophageal reflux disease (GERD) is a condition that develops when there is a retrograde flow of stomach contents back into the esophagus^1–3^. Long-term exposure to gastric contents may irritate the esophageal epithelium, leading to a spectrum of disease in three different phenotypes—non-erosive reflux disease (NERD), erosive esophagitis (EE), and Barrett’s esophagus (BE)—when inspected through endoscopy and/or histopathology^4–6^. Typical clinical presentations of GERD are heartburns and regurgitation, with atypical clinical presentations, such as epigastric pain, odynophagia, dysphagia, nausea, chronic cough, dental erosion, laryngitis, and asthma^7,8^.

Approximately 30% of GERD cases may progress to EE, and 1–13% of EE cases may also continue to develop BE^6^. However, reports of EE cases around the globe remain unclear, yet experts estimate the number hits approximately 1% of the population^7^. Aside from the burden on quality of life^9^, prolonged esophagitis may further induce esophageal epithelium metaplasia and progression of adenocarcinoma^10^. Due to its long-term morbidity, it is crucial to identify clear-cut risk factors that contribute to the development of EE to decide the need for endoscopy and/or histopathology analysis, to detect an early mucosal erosion, and to prevent its progression to BE and esophageal adenocarcinoma.

Given the burden on health-related quality of life, it is important for physicians to provide proper management and care from well-established knowledge of EE risk factors. Therefore, this meta-analysis aims to outline the detailed risk factors contributing to the development of EE as the primary outcome from the perspective of demography, comorbidities, and medication history. Furthermore, a secondary outcome of the global, regional, and local prevalence will also be depicted in this study since the exact number of cases reported is still unclear.

## Results

### Overview of literature search and included studies

**Figure 1:**
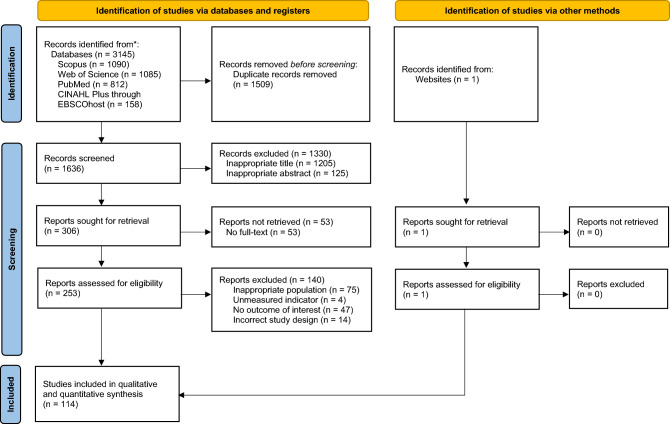
PRISMA flow diagram of the study selection process.

The initial search yields a total of 3145 studies, out of which, 1636 studies are removed due to duplication of studies. We obtain 306 studies with eligible titles and abstracts and review 253 studies, as the full-texts of 53 studies are irretrievable. Finally, only 114 eligible studies with a total of 759,100 participants are included in this study. The overall process is illustrated in Fig. 1. The summary of qualitative synthesis of the included studies is provided in Table 1.

**Table 1:** Basic characteristic of the included studies.

Author	Study location	Study design	EE diagnostic guideline	Age (mean ± SD)	Population characteristic and/or UGI endoscopy indication	Sample size	
						EE	Non-EE
Abraham et al.^26^	New York, USA	Case–control study	LA classification	52.74 ± 15.17	N/A	661	1590
Adekanle et al.^63^	Ile-Ife, Nigeria	Case–control study	LA classification	50.60 ± 13.03	Diagnosed with dyspepsia and had diagnostic UGI endoscopy	80	80
Al Shammaa et al.^64^	Nasiriyah/Baghdad, Iraq	Cross-sectional study	LA classification	40.71 ± 5.69	Having alarm symptoms of GERD and unsatisfactory response to PPI trial	44	66
Avidan et al.^65^	Illinois, US	Case–control study	Authors' criteria	58.07 ± 12.39	Having symptoms or complaints suggestive of a gastrointestinal disease	1533	3428
Avidan et al.^66^	Illinois, US	Cross-sectional study	Authors' criteria	64.31 ± 8.53	Diagnosed with any type of arthritis	41	154
Avidan et al.^67^	Chicago, US	Cross-sectional study	Authors' classification	57.11 ± 11.99	Having GERD symptoms	330	314
Baeg et al.^33^	Seoul, Korea	Cross-sectional study	LA classification	51.65 ± 11.42	Medical check-up	948	9390
Barreda Costa et al.^68^	Lima, Peru	Case–control study	Authors' definition	48.5 ± 13.03	Medical check-up	140	140
Chang et al.^69^	Taichung, Taiwan	Case–control study	LA classification	50.55 ± 12.23	Medical check-up	180	652
Chen et al.^70^	Taipei, Taiwan	Prospective cohort study	LA classification	78.00 ± 16.50	Having NGT feeding converted to PEG feeding	9	38
Cheng et al.^71^	Taichung, Taiwan	Case–control study	LA classification	49.27 ± 12.14	Medical check-up	208	176
Cheng et al.^72^	Tainan, Taiwan	Prospective cohort study	LA classification	43.14	N/A	424	100
Chiba et al.^73^	Tokyo, Japan	Cross-sectional study	LA classification	33.96 ± 3.85	Medical check-up	728	4262
Chih et al.^48^	Cheng Kung, Taiwan	Cross-sectional study	LA classification	49.34 ± 12.06	Medical check-up	1463	5889
Cho et al.^17^	Seoul, Korea	Cross-sectional study	LA classification	54.5 ± 9.4	Medical check-up	320	4981
Choi et al.^74^	Goyang City, South Korea	Cross-sectional study	LA classification	47.39 ± 12.82	Medical check-up	1077	13,646
Chua et al.^47^	Taipei, Taiwan	Case–control study	LA classification	48.44 ± 11.78	Medical check-up	427	427
Chue et al.^11^	Sengkang, Singapore	Retrospective cohort study	LA classification	N/A	Undergoing LSG and medical check-up prior to LSG	29	35
Chung SJ et al.^44^	Seoul, Korea	Case–control study	LA classification	47.6 ± 11.1	Medical check-up	3539	3539
Chung H et al.^75^	Seoul, Korea	Retrospective cohort study	LA classification	53.5 ± 10.9	Medical check-up	66	639
Chung TH et al.^76^	Ulsan, South Korea	Cross-sectional study	LA classification	50.91 ± 6.4	Shipyard male workers undergoing medical check-up	530	5510
Chung TH et al.^14^	Seoul, South Korea	Retrospective cohort study	LA classification	50.13 ± 6.51	Medical check-up	276	6874
Deppe et al.^77^	Munich, Germany	Cross-sectional study	N/A	49.7 ± 15.1	Diagnosed with GERD	29	42
El-Serag et al.^78^	All VA hospital in USA	Case–control study	N/A	58.00 ± 14.18	N/A	92,860	101,366
El-Serag et al.^79^	Houston, USA	Cross-sectional study	LA classification	44.61 ± 10.22	Hospital employee undergoing medical check-up	40	124
El-Serag et al.^80^	Houston, Texas, US	Cross-sectional study	LA classification	44 ± 10	Hospital employee undergoing medical check-up	44	152
Filiberti et al.^81^	12 areas in Italy	Case–control study	LA classification	54.66 ± 14.46	Patients referred for endoscopic examination	462	619
Fujiwara et al.^82^	Osaka, Japan	Cross-sectional study	LA classification	58.93 ± 13.35	Having GERD symptoms	164	89
Gaddam et al.^83^	Kansas, Missouri	Cross-sectional study	LA classification	56.87 ± 17.25	Having GERD symptoms	241	455
Gado et al.^84^	Giza, Egypt	Cross-sectional study	Savary-Miller classification	45 ± 15	N/A	106	327
Gatoupulou et al.^85^	Alexandroupolis, Greece	Cross-sectional study	LA classification	49.9	N/A	21	29
Gunji et al.^86^	Tokyo, Japan	Cross-sectional study	LA classification	51.7 ± 8.1	Medical check-up	1831	8009
Ha et al.^50^	Seoul, South Korea	Cross-sectional study	LA classification	45.06 ± 12.05	Medical check-up	292	500
Ham et al.^87^	Seoul, South Korea	Case–control study	ROME IV criteria of esophageal disorder	49.57 ± 14.62	Having GERD symptoms followed by impedance-pH testing	25	135
Heo et al.^88^	Seoul, South Korea	Cross-sectional study	LA classification	50.01 ± 7.95	Medical check-up	2316	29,027
Hsieh et al.^46^	Changhua, Taiwan	Cross-sectional study	LA classification	50.1 ± 11.2	Medical check-up	2916	1979
Hsu et al.^89^	Taipei, Taiwan	Cross-sectional study	LA classification	51.71 ± 11.52	Medical check-up	131	612
Hung WC et al.^45^	Cheng Kung, Taiwan	Cross-sectional study	LA classification	48.93 ± 12.96	Medical check-up	1922	10,168
Hung HH et al.^90^	Taipei, Taiwan	Retrospective cohort study	LA classification	52.07 ± 13.11	Medical check-up	4044	30,302
Hung WC et al.^91^	Tainan City, Taiwan	Cross-sectional study	LA classification	48.79 ± 12.92	Medical check-up	1922	10,168
Isshi et al.^92^	Tokyo, Japan	Prospective cohort study	LA classification	57.5 ± 13.9	Diagnosed with GERD and prescribed with PPI in routine clinical care	183	107
Jo et al.^93^	Seoul, South Korea	Cross-sectional study	LA classification	57.2 ± 12.4	Having GERD symptoms	62	64
Jonaitis LV et al.^94^	Kaunas, Lithuania	Cross-sectional study	LA classification	41.59 ± 12.27	Diagnosed with GERD	53	51
Jonaitis L et al.^95^	Kaunas, Lithuania	Prospective cohort study	Savary-Miller classification	38.29 ± 11.05	Diagnosed with *H. pylori*-positive duodenal ulcer	19	131
Jones et al.^29^	Chicago, Illinois, USA	Case–Control study	Hetzel-Dent classification	43.40 ± 11.84	Having GERD symptoms	26	49
Jones et al.^96^	Chicago, Illinois, USA	Case–control study	Hetzel-Dent classification	41.45 ± 12.57	Having GERD symptoms responsive to PPI therapy	19	28
Jung et al.^97^	Goyang, South Korea	Cross-sectional study	LA classification	52.9 ± 9.3	Medical check-up	50	246
Kainuma et al.^98^	Ishigaki City, Okinawa	Case–control study	LA classification	55.01 ± 11.90	Undergoing gastric cancer screening	35	218
Kang et al.^19^	Seoul, South Korea	Cross-sectional study	LA classification	43.9 ± 8.8	Medical check-up	161	2281
Kavitt et al.^99^	Nashville, USA	Prospective cohort study	LA classification	54.00 ± 11.72	Having GERD symptoms	11	30
Kawai et al.^100^	Tokyo, Japan	Cross-sectional study	LA classification	39.2 ± 8.4	Medical check-up	82	336
Kim HY^101^	Seongnam-si, South Korea	Case–control study	LA classification	55.4 ± 8.7	Medical check-up	239	968
Kim JG et al.^102^	Incheon, South Korea	Cross-sectional study	LA classification	48.39 ± 13.55	Having GERD and/or dyspeptic symptoms	80	89
Kim JY et al.^103^	Seoul, South Korea	Case–control study	LA classification	50.72 ± 13.57	Medical check-up	70	147
Kim SY et al.^23^	Seoul, South Korea	Retrospective cohort study	LA classification	47.1 ± 10.2	Medical check-up	651	9507
Ko et al.^18^	Seoul, South Korea	Cross-sectional study	LA classification	43.56 ± 12.29	Medical check-up	449	2543
Koo et al.^104^	Ansan, South Korea	Prospective cohort study	LA classification	43.73 ± 9.89	Medical check-up	42	987
Kulig et al.^13^	Germany, Austria, and Switzerland	Prospective cohort study	Modified LA classification	53.48 ± 14.01	Having GERD symptoms	2660	2853
Lee D et al.^105^	Suwon-si, South Korea	Case–control study	LA classification	45.03 ± 8.54	Medical check-up	675	8165
Lee YC et al.^106^	Taipei, Taiwan	Cross-sectional study	LA classification	52.2 ± 12	Medical check-up	843	3757
Lee HL et al.^107^	Seoul, South Korea	Case–control study	LA classification	45.45 ± 11.77	Medical check-up	292	2896
Lee HL et al.^108^	Seoul, South Korea	Case–control study	LA classification	51.69 ± 10.29	N/A	100	100
Lee ES et al.^109^	Seoul, South Korea	Prospective cohort study	LA classification	54.3 ± 13.5	Having GERD symptoms	205	200
Lee SD et al.^110^	Seoul, South Korea	Cross-sectional study	LA classification	59.3 ± 9.1	Having T2DM for ≥ 5 years	18	77
Lee SW et al.^111^	Taichung, Taiwan	Cross-sectional study	N/A	46.16 ± 15.44	Diagnosed with GERD	87	86
Lee SW et al.^112^	Taipei, Taiwan	Case–control study	N/A	45.58 ± 14.46	Diagnosed with GERD	87	174
Lee SW et al.^113^	Taichung, Taiwan	Cross-sectional study	N/A	52.36 ± 11.86	Medical check-up	1118	6499
Lee SW et al.^114^	Taichung, Taiwan	Case–control study	N/A	54.30 ± 13.01	Medical check-up	100	100
Lee H et al.^115^	Seoul, South Korea	Retrospective cohort study	LA classification	50.47 ± 8.17	Medical check-up	1367	10,319
Li et al.^116^	Taipei, Taiwan	Case–control study	LA classification	52.39 ± 11.59	Medical check-up	166	507
Lien et al.^117^	Taichung, Taiwan	Cross-sectional study	Modified Savary-Miller classification	49.80 ± 12.05	Medical check-up	342	3544
Lippmann et al.^118^	North Carolina, US	Case–control study	N/A	49.74 ± 13.73	N/A	72	289
Loke et al.^39^	Kaohsiung, Taiwan	Case–control study	LA classification	51.2 ± 11.2	Medical check-up	507	507
Lord et al.^119^	Los Angeles, California, USA	Cross-sectional study	Modified Savary-Miller classification	48.52 ± 14.30	Having GERD symptoms and had been treated with laparoscopic Nissen fundoplication	77	39
Mahdi et al.^120^	Baghdad, Iraq	Case–control study	Savary-Miller classification	44.63 ± 12.50	Diagnosed with GERD	60	100
Matsuda et al.^121^	Tokyo, Japan	Cross-sectional study	LA classification	56.6 ± 14.6	Medical check-up and having SSc as the main case group	20	46
Matsuki et al.^122^	Osaka, Japan	Case–control study	LA classification	52.81 ± 10.76	Medical check-up	138	713
Matsumura et al.^123^	Chiba, Japan	Prospective cohort study	LA classification	56.53 ± 15.34	Having GERD symptoms	24	96
Meira et al.^124^	Brumado, Bahia	Cross-sectional study	LA classification	44.11 ± 15.34	Having GERD symptoms	281	395
Migaczewski et al.^125^	Krakow, Poland	Prospective cohort study	N/A	39.26 ± 11.15	Severely obese patients (BMI > 40 kg/m2 or > 35 kg/m2 with obesity-related comorbidities) undergoing LSG	9	13
Minatsuki et al.^126^	Chiba, Japan	Cross-sectional study	LA classification	50.85 ± 9.33	Medical check-up	733	10,104
Mun et al.^15^	Seoul, South Korea	Cross-sectional study	LA classification with Japanese modification	36.35 ± 8.15	Medical check-up	49,767	197,683
Nam et al.^127^	Seoul, South Korea	Cross-sectional study	LA classification	49.8 ± 9.6	Medical check-up	838	10,852
Noh et al.^128^	Seoul, Korea	Cross-sectional study	LA classification	42.75 ± 8.74	Medical check-up	286	2102
Nurleili et al.^12^	Jakarta, Indonesia	Cross-sectional study	LA classification	N/A	Having GERD symptoms	31	25
Ohashi et al.^129^	Kyoto, Japan	Cross-sectional study	LA classification	52.74 ± 9.96	Medical check-up	118	315
Oikawa et al.^130^	Sendai, Japan	Case–control study	LA classification	62.38 ± 12.17	Diagnosed with *H. pylori*-positive	110	202
Ou et al.^51^	Kaohsiung, Taiwan	Cross-sectional study	LA classification	51.4 ± 12.19	Medical check-up	352	1688
Park JH et al.^49^	Seoul, Korea	Case–control study	LA classification	45.19 ± 9.3	Medical check-up	1679	3358
Park CH et al.^131^	Seoul, South Korea	Case–control study	LA classification	46.76 ± 13.02	Medical check-up	742	1484
Rafat et al.^132^	Cairo, Egypt	Case–control study	LA classification	47.85 ± 11.64	Having GERD symptoms	90	40
Ronkainen et al.^133^	Kalix and Haparanda, Sweden	Cross-sectional study	LA classification	53.35	N/A	155	769
Sadiku et al.^16^	Durrës, Albania	Case–control study	LA classification	46.45 ± 16.13	Having GERD symptoms	248	273
Savarino et al.^134^	Genoa, Italy	Case–control study	LA classification	48.74 ± 14.41	Having GERD symptoms	58	168
Savarino et al.^135^	Genoa, Italy	Cross-sectional study	LA classification	48.08 ± 10.81	Having GERD symptoms	81	295
Shaker et al.^136^	Zagazig, Egypt	Cross-sectional study	LA classification	54.7 ± 7.1	Having GERD symptoms	65	71
Shimamoto et al.^137^	Kamogawa, Japan	Cross-sectional study	LA classification	50.4 ± 8.8	Medical check-up	994	5901
Shimatani et al.^138^	Hiroshima, Japan	Case–control study	LA classification	67.98 ± 17.19	N/A	65	68
Sogabe et al.^139^	Kagawa, Japan	Cross-sectional study	LA classification	56.9 ± 8.4	Male with MetS undergoing medical check-up	55	210
Sogabe et al.^140^	Shikokucho, Japan	Cross-sectional study	LA classification	53.7 ± 9.2	Medical check-up	1348	5749
Tai et al.^141^	Kaohsiung, Taiwan	Cross-sectional study	LA classification	31.49 ± 9.90	Severely obese patients (BMI ≥ 32 kg/m2)	84	176
Tai et al.^142^	Kaohsiung, Taiwan	Cross-sectional study	LA classification	37.2 ± 12.7	Severely obese patients (BMI ≥ 37 kg/m2 or ≥ 32 kg/m2 with obesity-related comorbidities) undergoing LSG	44	22
Vaishnav et al.^143^	Maharashtra, India	Cross-sectional study	LA classification	46 ± 10.6	Having dyspeptic symptoms for ≥ 2 mos and referred for gastroscopy	91	85
Wang FW et al.^144^	Kaohsiung, Taiwan	Cross-sectional study	LA classification	51.5 ± 12.9	Medical check-up	70	502
Wang PC et al.^145^	Hualien, Taiwan	Cross-sectional study	LA classification	52.08 ± 11.38	Medical check-up	86	508
Wang K et al.^146^	Anyang, China	Cross-sectional study	LA classification	57.36 ± 6.96	High esophageal SCC prevalent area	271	2573
Wei et al.^147^	New Taipei, Taiwan	Cross-sectional study	LA classification	51.57 ± 10.21	Medical check-up	427	1410
Wu et al.^148^	Wuhan, China	Case–control study	LA classification	46.71 ± 1.13	N/A	182	190
Wu et al.^149^	Shanghai, China	Case–control study	LA classification	49.70 ± 1.47	Medical check-up	268	269
Yamamoto et al.^150^	Tokyo, Japan	Case–control study	LA classification	69.55 ± 12.08	Taking low-dose aspirin 100 mg/day for ≥ 1 mo	25	293
Yang et al.^151^	Tainan City, Taiwan	Prospective cohort study	LA classification	43.8	Diagnosed with *H. pylori*-positive duodenal ulcer	57	293
Yasuhara et al.^152^	Kanonji, Japan	Cross-sectional study	LA classification	50.49 ± 7.54	Company employee undergoing medical check-up	127	1368
Ye et al.^153^	Nanjing, China	Cross-sectional study	LA classification	47.6 ± 13.1	Having GERD symptoms	308	282
Ze et al.^154^	Seoul, South Korea	Cross-sectional study	LA classification	47.15 ± 8.35	Medical check-up	65	663

BMI, body mass index; GERD, gastroesophageal reflux disease; LA, Los Angeles; LSG, laparoscopic sleeve gastrectomy; MetS, metabolic syndrome; N/A, not available; NGT, nasogastric tube; PEG, percutaneous endoscopic gastrectomy; PPI, proton pump inhibitor; SCC, squamous cell carcinoma; SSc, systemic sclerosis; T2DM, type 2 diabetes mellitus; UGI, upper gastrointestinal.

Approximately 25.53% of participants are diagnosed as EE through upper gastrointestinal (UGI) endoscopy. The mean age is 47.56 years; two studies did not report the mean age of their study population^11,12^. To avoid proportional bias, we cannot report the gender proportion because 28 studies are missing this information. Among the 114 included studies, 36 are case–control, 11 are prospective cohort, 6 are retrospective cohort, and 61 are cross-sectional studies. In terms of regions, 84 studies are in Asia, 15 studies in America, 11 studies in Europe, and 4 studies in Africa.

### Demographical factors

The demographical factors chosen for this analysis are as follows: sex, age, race, employment status, marital status, educational status, educational duration, and disease duration (Table 2). The forest and funnel plots are provided in Supplementary Fig. S1–S8 online. Evidence of high heterogeneity is detected in sex (I^2^ = 77%), age (I^2^ = 96%), race (I^2^ = 71%), employment status (I^2^ = 91%), and educational status (I^2^ = 85%). All heterogeneity tests are performed using REM. Four factors are found as risk factors: (1) Age ≥ 60 y.o. with OR 2.03 (95% CI = 1.81–2.28, n = 92 studies); (2) White/Caucasian race with OR 1.67 (95% CI = 1.40–1.99, n = 10 studies); (3) Being single with OR 1.08 (95% CI = 1.03–1.14, n = 7 studies); and (4) Having GERD ≥ 5 years with OR 1.27 (95% CI = 1.14–1.42, n = 2 studies). We define ‘having GERD ≥ 5 years’ as having symptomatic GERD that is not diagnosed by endoscopy for 5 years or more. The rest – being male, employed workers, being students of college or higher educational degree, and study duration ≥ 12 years—are not risk nor protective factors.

**Table 2:** Forest plot results of the demographical factors, comorbidities, and medication history.

Factors	Definition	Number of studies	IV odds ratio (95% CI)	Analysis model	Heterogeneity		Overall effect		Egger's test	
					p-value	I^2^ (%)	Z-score	p-value	**Z-score**	**p-value**
Demographical factors										
Sex	Male vs. Female	17	1.01 (0.88–1.17)	REM	** < 0.00001**	77	0.16	0.87	-0.13	0.90
Age	Age ≥ 60 y.o	92	2.03 (1.81–2.28)	REM	** < 0.00001**	96	11.91	** < 0.00001**	0.97	0.33
Race	White / Caucasian vs. Non-White / Non-Caucasian	10	1.67 (1.40–1.99)	REM	**0.0003**	71	5.71	** < 0.00001**	-0.58	0.56
Employment status	Employed vs. Unemployed	2	0.76 (0.39–1.48)	REM	**0.0008**	91	0.81	0.42	-0.67	0.50
Marital status	Single vs. Married	7	1.08 (1.03–1.14)	REM	0.38	7	2.94	**0.003**	-0.39	0.70
Educational status	College or higher vs. Others	7	0.97 (0.76–1.24)	REM	** < 0.00001**	85	0.25	0.8	-0.16	0.88
Educational duration	Study ≥ 12 years	3	1.00 (0.86–1.15)	REM	0.36	3	0.04	0.97	1.16	0.25
Disease duration	Having GERD ≥ 5 years	2	1.27 (1.14–1.42)	REM	0.73	0	4.23	** < 0.0001**	-0.2	0.84
Comorbidities										
General obesity	Based on BMI with multiple cut-offs from 25 to 40 kg/m^2^ for M and F	50	1.78 (1.61–1.98)	REM	** < 0.00001**	85	10.93	** < 0.00001**	1.22	0.22
Central obesity	Based on WC with multiple cut-offs: • From 85 to 102 cm for M • From 80 to 88 cm for F	25	1.29 (1.18–1.42)	REM	** < 0.00001**	69	5.44	** < 0.00001**	2.03	**0.04**
DM/hyperglycemia	Based on several criteria: • Having history or medication of DM • ADA diagnostic criteria • FBG with multiple cut-offs from 100 to 126 mg/dL • HbA1c ≥ 6.5%	38	1.24 (1.17–1.32)	REM	**0.04**	30	6.68	** < 0.00001**	0.2	0.84
Hypertension/elevated BP	Based on several criteria: • Having history or medication of hypertension • SBP/DBP with cut-offs, either 130/85 or 140/90	36	1.16 (1.09–1.23)	REM	**0.0006**	48	4.62	** < 0.00001**	0.93	0.35
Dyslipidemia	Based on history of dyslipidemia	10	1.15 (1.06–1.24)	REM	0.42	3	3.31	**0.0009**	-0.88	0.38
Hypertriglyceridemia	TG ≥ 150 mg/dL	22	1.42 (1.29–1.57)	REM	** < 0.00001**	70	6.9	** < 0.00001**	1.85	0.06
Hypercholesterolemia	TC ≥ 200 mg/dL	4	1.51 (0.95–2.40)	REM	**0.03**	67	1.73	0.08	1.23	0.22
High LDL-C	LDL-C ≥ 130 mg/dL	3	1.37 (0.64–2.94)	REM	**0.04**	69	0.81	0.42	2.16	**0.03**
Low HDL-C	HDL-C < 40 (M) or < 50 (F) mg/dL	17	1.04 (0.95–1.13)	REM	**0.001**	56	0.82	0.41	-2.23	**0.03**
Hiatal hernia	Based on endoscopic findings	57	4.07 (3.21–5.17)	REM	** < 0.00001**	95	11.52	** < 0.00001**	0.28	0.78
*H. pylori* infection	Based on one or more positive diagnostic tools: • *H. pylori*-specific IgG antibody • Tissue biopsy with Giemsa, HE, or Warthin-Starry staining followed or not followed by culture • Rapid urease/CLO test • UBT	39	0.56 (0.48–0.66)	REM	** < 0.00001**	91	6.85	** < 0.00001**	1.45	0.15
Gastric ulcer	Based on endoscopic findings	7	0.83 (0.56–1.23)	REM	**0.0002**	77	0.91	0.37	-0.94	0.35
Duodenal ulcer	Based on endoscopic findings	6	0.94 (0.63–1.38)	REM	**0.03**	61	0.33	0.74	0.07	0.95
Atrophic gastritis	Based on endoscopic findings	8	0.51 (0.31–0.86)	REM	** < 0.00001**	84	2.51	**0.01**	0.02	0.99
NAFLD	Based on several criteria: • History of NAFLD • FibroScan findings of NAFLD • Abdominal USG findings of NAFLD	8	1.26 (1.18–1.34)	REM	0.68	0	7.27	** < 0.00001**	0.41	0.68
Medication history										
NSAID	N/A	11	1.02 (0.94–1.10)	REM	0.52	0	0.39	0.7	-1.05	0.29
Aspirin	N/A	8	1.09 (0.96–1.24)	REM	0.22	26	1.39	0.17	-1.27	0.20
NSAID and/or aspirin	The use of NSAID and/or aspirin were not separated by the included studies	3	1.21 (0.79–1.86)	REM	0.09	58	0.88	0.38	1.11	0.27
PPI	N/A	6	0.65 (0.30–1.39)	REM	** < 0.00001**	93	1.12	0.26	0.18	0.86
H2RA	N/A	3	1.23 (0.63–2.39)	REM	0.12	53	0.61	0.54	-0.52	0.61
Antacids	N/A	2	1.97 (0.98–3.93)	REM	**0.01**	84	1.91	0.06	0.94	0.34

ADA, American Diabetes Association; CI, confidence interval; CLO, *Campylobacter*-like organism; DBP, diastolic blood pressure; DM, diabetes mellitus; FBG, fasting blood glucose; GERD, gastroesophageal reflux disease; H2RA, histamine-2 receptor antagonist; HbA1c, hemoglobin A1c; HDL-C, high density lipoprotein cholesterol; HE, Hematoxilin-Eosin; IgG, immunoglobulin G; IV, inverse variance; LDL-C, low density lipoprotein cholesterol; N/A, not applicable; NAFLD, non-alcoholic fatty liver disease; NSAID, non-steroidal anti-inflammatory drug; PPI, proton pump inhibitor; REM, random-effect model; SBP, systolic blood pressure; TC, total cholesterol; TG, triglyceride; UBT, urea breath test; USG, ultrasonography; WC, waist circumference.

The sensitivity analysis on employment status shows that the pooled effect of EE in employed patients is changed from nonsignificant to significant after removing one study by Kulig et al.^13^. However, the pooled effects of two other factors in the leave-one-out sensitivity analyses are changed from significant to nonsignificant after removing one study, either Chung et al.^14^, Kulig et al.^13^, Mun et al.^15^, or Sadiku et al.^16^, for marital status, and Kulig et al.^13^ for disease duration. The sensitivity analyses of the remaining demographical factors suggest that the pooled effects are not influenced by any single study.

### Comorbidities

Fifteen comorbidities are included in this analysis consisting general obesity, central obesity, diabetes mellitus (DM) or hyperglycemia, hypertension or elevated blood pressure (BP), dyslipidemia, hypertriglyceridemia, hypercholesterolemia, high low density lipoprotein cholesterol (LDL-C), low high density lipoprotein cholesterol (HDL-C), hiatal hernia (HH), *H. pylori* infection, gastric ulcer, duodenal ulcer, atrophic gastritis, and non-alcoholic fatty liver disease (NAFLD) (Table 2). The forest and funnel plots are provided in Supplementary Fig. S9–S23 online. We detect moderate to high heterogeneity in 11 out of 15 comorbidities, including general obesity (I^2^ = 85%), central obesity (I^2^ = 69%), hypertriglyceridemia (I^2^ = 70%), hypercholesterolemia (I^2^ = 67%), high LDL-C (I^2^ = 69%), low HDL-C (I^2^ = 56%), HH (I^2^ = 95%), *H. pylori* infection (I^2^ = 91%), gastric ulcer (I^2^ = 77%), duodenal ulcer (I^2^ = 61%), and atrophic gastritis (I^2^ = 84%). All heterogeneity tests are performed using REM. Based on the ORs, eight comorbidities that can be considered risk factors are as follows: (1) general obesity with OR 1.78 (95% CI = 1.61–1.98, n = 50 studies); (2) central obesity with OR 1.29 (95% CI = 1.18–1.42, n = 25 studies); (3) DM or hyperglycemia with OR 1.24 (95% CI = 1.17–1.32, n = 38 studies); (4) hypertension or elevated BP with OR 1.16 (95% CI = 1.09–1.23, n = 36 studies); (5) dyslipidemia with OR 1.15 (95% CI = 1.06–1.24, n = 10 studies); (6) hypertriglyceridemia with OR 1.42 (95% CI = 1.29–1.57, n = 22 studies); (7) HH with OR 4.07 (95% CI = 3.21–5.17, n = 57 studies); and (8) NAFLD with OR 1.26 (95% CI = 1.18–1.34, n = 8 studies). On the contrary, *H. pylori* infection (OR 0.56 [0.48–0.66]; n = 39 studies) and atrophic gastritis (OR 0.51 [0.31–0.86]; n = 8 studies) act as protective factors. Other factors – hypercholesterolemia, high LDL-C, low HDL-C, gastric ulcer, and duodenal ulcer – are not risk nor protective factors.

After removing a study by Cho et al.^17^ in the sensitivity analysis of duodenal ulcer, the pooled effect is shifted from nonsignificant to significant. On the contrary, the pooled OR of atrophic gastritis is shifted from significant to nonsignificant following the removal of a study by Ko et al.^18^. The leave-one-out sensitivity analyses of the remaining comorbidities suggest that the provided overall effects are robust and not affected by any single study.

### Medication history

We include five pharmacological medications: Non-steroidal anti-inflammatory drug (NSAID) only, aspirin only, NSAID and/or aspirin, proton pump inhibitor (PPI), H2 receptor antagonist (H2RA), and antacids (Table 2). The forest and funnel plots are provided in Supplementary Fig. S24–S29 online. NSAID and/or aspirin (I^2^ = 58%) and H2RA (I^2^ = 53%) have moderate heterogeneity, while PPI (I^2^ = 93%) and antacids (I^2^ = 84%) have high heterogeneity. All heterogeneity tests are performed using REM. There is no medication history considered as risk nor protective factors in the current analysis: NSAID only (OR 1.02 [0.94–1.10]), aspirin only (OR 1.09 [0.96–1.24]), NSAID and/or aspirin (OR 1.21 [0.79–1.86]), PPI (OR 0.65 [0.30–1.39]), H2RA (OR 1.23 [0.63–2.39]), and antacids (OR 1.97 [0.98–3.93]).

The sensitivity analysis of the antacids use reveals that the overall effect is changed from nonsignificant to significant following the removal of one study by Kang et al.^19^. On the other hand, no study has a notable influence in the leave-one-out sensitivity analyses of the remaining medication histories, proving the robustness of the pooled results.

### EE prevalence

**Figure 2:**
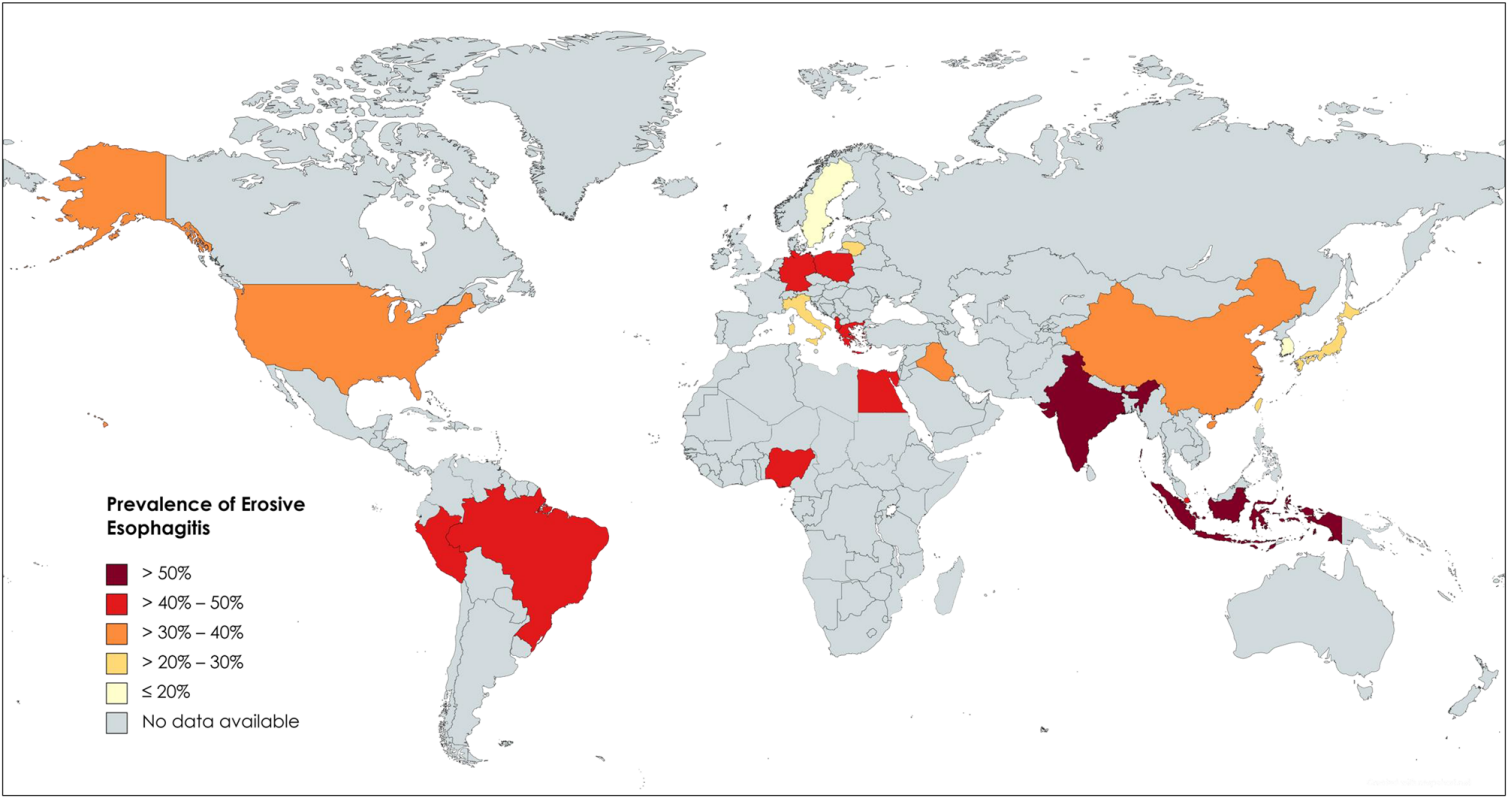
The distribution map of worldwide erosive esophagitis (EE) prevalence (created with https://www.mapchart.net/).

We perform meta-analysis of EE prevalence based on the geographic regions (Table 3) along with the substantial variations of the EE worldwide prevalence (Fig. 2 and Supplementary Fig. S30 online). There are 193,819 participants who are diagnosed with EE giving an overall pooled prevalence of 28% (95% CI = 24%–31%). The two highest pooled prevalence of EE are Africa (47% [95% CI = 27%–68%]) and the Middle East (43% [95% CI = 28%–60%]), while the lowest is Asia (24% [95% CI = 22%–27%]). Interestingly, the prevalence of EE in America (36% [95% CI = 30%–42%]) and Europe (34% [95% CI = 25%–44%]) are both higher than that in Asia. The top five countries in terms of prevalence are as follows: Indonesia (55% [95% CI = 42%–68%]), India (52% [95% CI = 44%–59%]), Nigeria (50% [95% CI = 42%–58%]), Peru (50% [95% CI = 44%–56%]), and Albania (48% [95% CI = 43%–52%]). The country with lowest pooled prevalence is Sweden (17% [95% CI = 15%–19%]).

**Table 3:** Worldwide pooled prevalence of EE based on geographical regions and countries.

Research area	Number of studies	Total subjects with EE	Total sample size	EE prevalence				Heterogeneity		
				Pooled value (%)	95% CI (%)	LCI	HCI	I^2^	*Cochran's Q*	p-value
Overall	114	193,819	759,100	0.28	0.24–0.31	0.24	0.31	99.89%	100,926.53	**0.00**
Geographic regions										
America	15	96,376	204,929	0.36	0.30–0.42	0.30	0.42	98.89%	1258.84	**0.00**
North America	13	95,955	203,973	0.34	0.27–0.41	0.27	0.41	99.04%	1249.74	**0.00**
South America	2	421	956	0.44	0.41–0.47	0.41	0.47	N/C	N/C	N/C
Africa	4	341	859	0.47	0.27–0.68	0.27	0.68	97.11%	103.63	**0.00**
Asia	84	93,307	544,274	0.24	0.22–0.27	0.22	0.27	99.72%	29,195.79	**0.00**
West Asia	2	104	270	0.39	0.33–0.44	0.33	0.44	N/C	N/C	N/C
East Asia	79	93,052	543,708	0.23	0.21–0.26	0.21	0.26	99.73%	28,953.34	**0.00**
Southeast Asia	2	60	120	0.50	0.41–0.59	0.41	0.59	N/C	N/C	N/C
South Asia	1	91	176	0.52	0.44–0.59	0.44	0.59	N/C	N/C	N/C
Europe	11	3795	9038	0.34	0.25–0.44	0.25	0.44	98.22%	561.34	**0.00**
Other region										
Middle East	5	365	969	0.43	0.28–0.60	0.28	0.60	95.78%	94.81	**0.00**
Countries										
Albania	1	248	521	0.48	0.43–0.52	0.43	0.52	N/C	N/C	N/C
Brazil	1	281	676	0.42	0.38–0.45	0.38	0.45	N/C	N/C	N/C
China	4	1029	4343	0.39	0.13–0.69	0.13	0.69	99.66%	895.17	**0.00**
Egypt	3	261	699	0.47	0.20–0.74	0.20	0.74	N/C	N/C	N/C
Germany	1	29	71	0.41	0.30–0.52	0.30	0.52	N/C	N/C	N/C
Greece	1	21	50	0.42	0.29–0.56	0.29	0.56	N/C	N/C	N/C
India	1	91	176	0.52	0.44–0.59	0.44	0.59	N/C	N/C	N/C
Indonesia	1	31	56	0.55	0.42–0.68	0.42	0.68	N/C	N/C	N/C
Iraq	2	104	270	0.39	0.33–0.44	0.33	0.44	N/C	N/C	N/C
Italy	3	601	1683	0.30	0.16–0.45	0.16	0.45	N/C	N/C	N/C
Japan	18	6780	44,866	0.23	0.19–0.28	0.19	0.28	99.08%	1847.43	**0.00**
Lithuania	2	72	254	0.26	0.21–0.32	0.21	0.32	N/C	N/C	N/C
Nigeria	1	80	160	0.50	0.42–0.58	0.42	0.58	N/C	N/C	N/C
Peru	1	140	280	0.50	0.44–0.56	0.44	0.56	N/C	N/C	N/C
Poland	1	9	22	0.41	0.23–0.61	0.23	0.61	N/C	N/C	N/C
Singapore	1	29	64	0.45	0.34–0.57	0.34	0.57	N/C	N/C	N/C
South Korea	31	67,227	396,199	0.18	0.14–0.21	0.14	0.21	99.83%	18,096.01	**0.00**
Sweden	1	155	924	0.17	0.15–0.19	0.15	0.19	N/C	N/C	N/C
Taiwan	26	18,016	98,300	0.29	0.24–0.35	0.24	0.35	99.68%	7835.90	**0.00**
US	13	95,955	203,973	0.34	0.27–0.41	0.27	0.41	99.04%	1249.74	**0.00**

CI, confidence interval; EE, erosive esophagitis; HCI, higher confidence interval; LCI, lower confidence interval.

### Publication bias and quality assessment

The funnel plots of central obesity (Supplementary Fig. S10B online), high LDL-C (Supplementary Fig. S16B online), and low HDL-C (Supplementary Fig. S17B online) show an asymmetrical distribution of studies, revealing the potential of publication bias. These findings are further confirmed by significant Egger’s test result in each factor (Z = 2.03 and p = 0.04 for central obesity, Z = 2.16 and p = 0.03 for high LDL-C, Z = -2.23 and p = 0.03 for low HDL-C). On the contrary, no potential of publication bias is found in the rest of the factors since their funnel plots show a rather symmetrical distribution of studies, further supported by their insignificant Egger’s test results (Table 2).

The quality of each study is shown in Table S1–S3. The overall quality of the included case–control studies (Supplementary Table S1 online) is good in 27 studies, while the rest (n = 9) is moderate. Of the 17 cohort studies, thirteen and four studies have good- and moderate-quality, respectively (Supplementary Table S2 online). The qualities of 61 cross-sectional studies (Supplementary Table S3 online) are as follows: (1) very good for 37 studies; (2) good for 19 studies; and (3) satisfactory for 5 studies. There are no poor-quality and unsatisfactory studies in the current meta-analysis.

## Discussion

To the best of our understanding, this meta-analysis is the first to thoroughly analyze the risk factors and prevalence of EE across the world from 1997 to 2021. Our results indicate that several demographical factors—age ≥ 60 y.o., White/Caucasian, single or unmarried, and having GERD ≥ 5 years—increase the risk of having EE. Interestingly, we find both risk and protective factors towards EE in the comorbidities. Obesity, DM, hypertension, dyslipidemia, hypertriglyceridemia, HH, and NAFLD are found to increase the risk of EE, while *H. pylori* infection and atrophic gastritis are found to be protective towards EE. Our results also indicate that medication history is not significantly increasing the risk nor protective of EE. The prevalence of EE in each of America, Africa, and Europe is higher than that in Asia and the highest prevalence is found to be in Africa and the Middle East.

Our study indicates that the risk of EE in males is twice than that in females. Previous studies have suggested that the combination of behavioral, immunologic, and metabolic aspects, especially in men, can increase the risk of EE and affect its prevalence. For example, Erol and Karpyak^20^ and Matsuzaki et al.^21^ suggest that cigarette smoking and alcohol consumption are more common in men and may increase the risk of having EE in men, approximately two to three times more than women. A longitudinal study by Adachi et al.^22^ also indicates that the prevalence of EE in men during 10-year period is increasing mainly due to aging, high BMI, and large diaphragmatic hiatus. This change, however, is not found in women. Furthermore, previous studies by Yoon Kim et al.^23^ and Sun Kim et al.^24^ suggest the protective effects of estrogen, although the studies use animal models.

Our study shows that the risk of EE in the Western (White/Caucasian) population is approximately two-fold higher than that in the non-White/Caucasian population. Previous studies have suggested that lifestyle factors, anatomical, and genetic variance can also explain the high risk of EE in the Western population. In terms of lifestyle factors, Wirth et al.^25^, Abraham et al.^26^, and Ko et al.^18^ indicate the differences in the risk can be attributed to the differences in eating habits or cultures (e.g. high fat diet and alcohol drinking in the Western population), distribution of visceral fat tissues, and body composition between the Western and Eastern populations^25,26^. In terms of anatomical differences, previous studies also suggest that the mass of gastric parietal cells of Western population is greater than that in the Asian population, which explains the higher gastric acid production in the Western population^18,25^. Moreover, in terms of genetic variance, some previous studies indicate that the difference in the ABH-secretor and Lewis histo-blood group may explain the difference of risk in the Western population. In particular, Wirth et al.^25^ and Suzuki et al.^27^ indicate that individuals with group A and non-secretors (common in the Western population) are prone to have EE.

This study finds that HH increases the risk of EE and this may be explained by anatomical and physiological factors. HH may diminish the augmenting effect of diaphragmatic crus to prevent gastric reflux^28^. Previous study mentions that the size of the HH is the most important risk factor of EE in individuals with GERD^29^. Some etiologies, such as pregnancies, surgical history, being elderly, and overweight, may increase the probability of HH^30,31^.

Obese individuals tend to experience more frequent and intense reflux symptoms compared to non-obese individuals^32^. Anatomically, obesity may promote esophagitis by increasing intra-abdominal pressure (IAP) and inducing lower esophageal sphincter (LES) relaxation^33^. Another evidence also reveals that obesity increases the transvesically-measured IAP^34^. Another mechanism thought to be involved in EE is related to adipose tissue. It may act as an endocrine tissue releasing inflammatory cytokines and leptin, which may further exacerbate the esophageal inflammatory process^35^.

In terms of metabolic diseases other than obesity, DM may cause esophageal dysfunction, which results in the amplitude reduction of esophageal contractions, less peristaltic waves, decreased LESP (lower esophageal sphincter pressure), and abnormal gastroesophageal reflux^36,37^. This is consistent with our finding that the risk of EE is increased in diabetic patients. Interestingly, the esophageal dysfunction in diabetic patients is also associated with autonomic neuropathy involving the vagal nerve, especially when the patient is in hyperglycemic state or has diabetes for 5–10 years after onset^38,39^. Gastric emptying can be disrupted due to this process, which triggers EE^39^. This process is further worsened by the fact that reflux symptoms may be more frequent in diabetic patients with three major complications (retinopathy, neuropathy, nephropathy) and longer duration of DM^40,41^.

In this study, we find that hypertension increases the risk of EE. This finding is first confirmed by Gudlaugsdottir et al.^42^, which finds a significantly higher systolic blood pressure (SBP) in EE compared to the controls, although the underlying pathophysiology is still unclear. The relationship between hypertension and esophageal reflux is further confirmed by Hu et al.^43^, which observes a significant improvement in the hypertension control after laparoscopic fundoplication during a 3.5 year follow-up period.

Our overall analysis finds dyslipidemia to be a risk factor for EE. However, most studies included in the analysis do not find dyslipidemia to be a risk factor. To evaluate this finding, we also separately analyzed several components of dyslipidemia, such as hypercholesterolemia, hypertriglyceridemia, high LDL-C, and low HDL-C. Our results suggest that hypertriglyceridemia is a risk factor of EE, but not dyslipidemia and its other components. Several studies have suggested triglyceride (TG) as an independent risk factor for EE related to humoral components that altered LESP and the frequency of transient relaxation^44,45^. TG has also been correlated with high fat intake, causing delayed gastric emptying time^46–48^. Moreover, hypertriglyceridemia is a significant predictive factor of EE severity, possibly related to fatty liver and insulin resistance^49^. The chronic inflammation in EE due to gastric acid injury may cause abnormal lipid metabolism, increasing TG^47^. Yet, several studies do not find TG to be an independent risk factor of EE^50,51^.

NAFLD also reaches statistical significance as a risk factor for EE. A study reports that only NAFLD is associated with EE, but not obesity^45^. NAFLD also increases the systemic oxidative stress and decreases the antioxidant capacity, which disrupts the gastric mucus layer and further causing esophageal mucosal damage and increasing the risk of EE^45^.

Interestingly, both gastric atrophy and *H. pylori* infection show to be protective factors for EE. The gastric atrophy can be classified into closed-type (C-type) and open type (O-type) according to the endoscopic atrophic border. According to Kim et al.^52^, the ambulatory pH monitoring study indicates that the O-type is associated with a lesser number of reflux symptoms and EE than the C-type. The O-type is characterized by an increasing number of impaired acid secreting parietal gastric cells will hinder more the gastric acid production, which will lead to hypochlorhydria, lessen the esophageal acidity, and further contribute to the pathogenesis of EE^52,53^. In a similar manner, the *H. pylori* infection may present protective mechanism since *H. pylori* chronic inflammation can cause gastric atrophy and further decreases the acid secretory capacity of the gastric lining^54,55^. It is only observable in O-type cases, while missing in the C-type, which produces higher gastrin and acid secretion^56^. However, this finding should be interpreted carefully since uneradicated *H. pylori* still carries a high risk of gastric cancer through several complex mechanisms^57^. Therefore, even though *H. pylori* is protective towards EE in our study, its eradication should still be well-considered to prevent the incidence of gastric cancer in later life.

To the best of our understanding, there has been no study that focuses on the meta-analysis of EE prevalence. We find that the prevalence of EE in America and Europe is higher than that in Asia. Recent meta-analyses on the prevalence of GERD^58^ and BE^59^ show similar results. A study by Qumseya et al.^60^ also finds a higher pooled prevalence of BE in low-risk Western populations compared to non-Western populations. One explanation for this distribution may be the difference in lifestyles. The typical Western diet is known to be high in fat, sodium, calories, and sugar, while it is low in fiber, fruits and vegetables. Concurrently, we have identified that White/Caucasian and individuals with obesity, type 2 diabetes mellitus, hypertension, dyslipidemia, and associated disease, such as NAFLD, are more significantly at risk of suffering from EE. Additionally, our meta-analysis shows a higher pooled prevalence of EE in Africa and the Middle East compared to those in other regions. This finding is in contrast to a previous BE meta-analysis by Eusebi et al.^59^, which finds the prevalence of BE in African and Middle Eastern countries to be lower than that in American countries.

We acknowledge several limitations in our study. First, we find some considerable high heterogeneities in most of the analyzed factors, mainly between the studies, such as population characteristics, various EE diagnostic criteria, differences in UGI study indications, and comorbidities along with various diagnostic criteria and cut-off values for their diagnosis. Second, although the EE diagnosis in the included studies is based on endoscopic result and the associated diagnostic criteria, endoscopy is still relatively an operator dependent-investigation, which may affect the EE prevalence in each country. Third, the number of included studies in several factors is still less than 10 studies; hence, the results should be carefully interpreted. Fourth, the included studies are mostly conducted in Asia (84 studies) and America (15 studies). This may affect the prevalence and risk factors of EE, and their interpretations in our study. Accordingly, we encourage more researchers from regions other than Asia to conduct more studies regarding the prevalence and risk factors of EE. However, regardless of the limitations, our study carries some strengths. The numbers of our included studies and their participants are relatively sufficient to cover a wide range of geographical areas; therefore, we can analyze the worldwide EE prevalence.

As the conclusion, we find several risk and protective factors of EE classified in three groups of factors, including demographical factors, comorbidities, and medication history. In the demographical factors, the risk of EE is increased due to age ≥ 60 y.o., being White/Caucasian, being single or unmarried, and having GERD ≥ 5 years. Interestingly, both risk and protective factors of EE are found in the comorbidities. Obesity, DM, hypertension, dyslipidemia, hypertriglyceridemia, HH, and NAFLD act as risk factors, while *H. pylori* infection and atrophic gastritis act as protective factors. The EE prevalence in each of America, Africa, and Europe are higher than that in Asia. Given these findings, an integrated care pathways of EE—including the decision regarding the timing of endoscopy based on the risk factors—is expected to be constructed, which then may help medical professionals to give proper and comprehensive managements for patients who are at a high risk of EE.

## Methods

This systematic review and meta-analysis were conducted in accordance with the Preferred Reporting Items for Systematic Reviews and Meta-Analyses (PRISMA) latest statement^61^. The protocol of this study has been previously registered to the International Prospective Register of Systematic Reviews (PROSPERO) database (CRD42023418716).

### Search strategy

A systematic computerized data searching of relevant studies was conducted in four electronic medical databases, including PubMed, Scopus, Cumulative Index to Nursing and Allied Health Literature (CINAHL) Plus database via EBSCOhost, and Web of Science, by two authors (A.P.W. and B.S.W.) from inception to June 2, 2022. The construction of keywords was performed based on Medical Subject Headings (MeSH) terms combined with their variance and other additional terms as following: “risk”, “predict”, “erosive esophagitis”, “gastroesophageal reflux disease”, and the variations of those terms. Boolean operators’ combinations were also applied in order to broaden and narrow the search results. The search was restricted to human participants only with no language and publication date restrictions.

### Eligibility criteria

The relevant studies were included if they met several following inclusion criteria: (1) study design of observational study; (2) study participants consisted of adult patients aged 18 years or older who had undergone upper gastrointestinal (UGI) endoscopy, either to screen or to diagnose EE; and (3) the measured outcomes were odds ratios (ORs) of any possible risk factors related to EE and number of EE events. The exclusion criteria were as follows: (1) duplicate studies; (2) irrelevant titles and/or abstracts; (3) irretrievable full-texts; and (4) incorrect study design (review articles, clinical trials, systematic reviews, meta-analyses, case reports or series, letter to editors, conference abstracts).

### Data extraction and quality assessment

All relevant studies were independently screened by seven of the co-authors. Any disagreements were resolved in a consensus involving all authors. The extracted data from the included studies were the author, year of publication, study location (country and region), study design, diagnostic guideline for EE, age, specific population characteristic, sample size, number of EE events, EE-related risk factors expressed in ORs, and the adjustment factors. We assessed the quality of the included studies using the Newcastle–Ottawa Scale (NOS) tool. For cohort and case–control studies, their quality was considered as good, moderate, or poor if their score was 7–9, 4–6, and 0–3, respectively. For cross-sectional studies, a score of 9–10 was considered as very good, 7–8 as good, 5–6 as satisfactory, and 0–4 as unsatisfactory. The quality assessment was conducted collaboratively through a group discussion by all authors, and the final decision was also taken based on the agreement of all authors.

### Statistical analysis

Meta-analyses were performed for the outcome of pooled ORs in each EE-related risk factor using RevMan ver. 5.4 (The Cochrane Collaboration, The Nordic Cochrane Centre, Copenhagen, Denmark). We also performed meta-analysis of pooled EE prevalence in each study using STATA ver. 16.0 (Stata Corporation, College Station, TX, USA) as the secondary outcome. The heterogeneity among studies was assessed using chi-square test (Cochran’s Q statistic). Then, we quantified the level of heterogeneity with the Higgins’ I^2^ statistic as follows: 0% was considered negligible heterogeneity, < 25% as low heterogeneity, 25–75% as moderate heterogeneity, and > 75% as high heterogeneity^62^. Since there was a considerable variability and diversity among studies and the characteristics of the study participants, we primarily applied the random-effect model (REM) for risk factors and prevalence analyses. P-value < 0.05 was considered statistically significant. The publication bias was visually assessed using funnel plot and quantitatively assessed using Egger’s test. Sensitivity analysis was carried out using the leave-one-out method.

### Supplementary Information

Supplementary Information 1.

Supplementary Figures.

Supplementary Tables.

## Data Availability

All data generated or analyzed during this study are included in this article and its supplementary material files. Further enquiries can be directed to the corresponding author.
