# Supplementary material for: Risk factors and 26-years worldwide prevalence of endoscopic erosive esophagitis from 1997 to 2022: a meta-analysis
Source: Sci Rep. 2023 Sep 14;13:15249. doi: 10.1038/s41598-023-42636-7 (PMC10502104; doi:10.1038/s41598-023-42636-7)
Supplement: Supplementary file 2 — Supplementary Figures. [file 41598_2023_42636_MOESM2_ESM.docx]

**A B**

**Supplementary Figure S1.** Odds ratio (OR) of being male (vs. female) related to the risk of erosive esophagitis (EE) in patients undergoing endoscopy. **(A)** Forest plot, **(B)** funnel plot. CI, confidence interval; IV, inverse variance; SE, standard error.

**A B**

**Supplementary Figure S2.** Odds ratio (OR) of age ≥60 y.o. (vs. <60 y.o.) related to the risk of erosive esophagitis (EE) in patients undergoing endoscopy. **(A)** Forest plot, **(B)** funnel plot. CI, confidence interval; IV, inverse variance; SE, standard error.

**A**

**B**

**Supplementary Figure S3.** Odds ratio (OR) of White / Caucasian race (vs. non-White / non-Caucasian race) related to the risk of erosive esophagitis (EE) in patients undergoing endoscopy. **(A)** Forest plot, **(B)** funnel plot. CI, confidence interval; IV, inverse variance; SE, standard error.

**A**

**B**

**Supplementary Figure S4.** Odds ratio (OR) of employment status (employed vs. unemployed) related to the risk of erosive esophagitis (EE) in patients undergoing endoscopy. **(A)** Forest plot, **(B)** funnel plot. CI, confidence interval; IV, inverse variance; SE, standard error.

**A**

**B**

**Supplementary Figure S5.** Odds ratio (OR) of marital status (single vs. married) related to the risk of erosive esophagitis (EE) in patients undergoing endoscopy. **(A)** Forest plot, **(B)** funnel plot. CI, confidence interval; IV, inverse variance; SE, standard error.

**A**

**B**

**Supplementary Figure S6.** Odds ratio (OR) of educational status (college or higher vs. others) related to the risk of erosive esophagitis (EE) in patients undergoing endoscopy. **(A)** Forest plot, **(B)** funnel plot. CI, confidence interval; IV, inverse variance; SE, standard error.

**A**

**B**

**Supplementary Figure S7.** Odds ratio (OR) of studying ≥12 years at any educational institutions (vs. <12 years) related to the risk of erosive esophagitis (EE) in patients undergoing endoscopy. **(A)** Forest plot, **(B)** funnel plot. CI, confidence interval; IV, inverse variance; SE, standard error.

**A**

**B**

**Supplementary Figure S8.** Odds ratio (OR) of having gastroesophageal reflux disease (GERD) ≥5 years (vs. <5 years) related to the risk of erosive esophagitis (EE) in patients undergoing endoscopy. **(A)** Forest plot, **(B)** funnel plot. CI, confidence interval; IV, inverse variance; SE, standard error.

**A B**

**Supplementary Figure S9.** Odds ratio (OR) of general obesity (based on body mass index [BMI]) related to the risk of erosive esophagitis (EE) in patients undergoing endoscopy. **(A)** Forest plot, **(B)** funnel plot. CI, confidence interval; IV, inverse variance; SE, standard error.

**A**

**B**

**Supplementary Figure S10.** Odds ratio (OR) of central obesity (based on waist circumference [WC]) related to the risk of erosive esophagitis (EE) in patients undergoing endoscopy. **(A)** Forest plot, **(B)** funnel plot. CI, confidence interval; IV, inverse variance; SE, standard error.

**A B**

**Supplementary Figure S11.** Odds ratio (OR) of diabetes mellitus (DM) or hyperglycemia related to the risk of erosive esophagitis (EE) in patients undergoing endoscopy. **(A)** Forest plot, **(B)** funnel plot. CI, confidence interval; IV, inverse variance; SE, standard error.

**A**

**B**

**Supplementary Figure S12.** Odds ratio (OR) of hypertension or elevated blood pressure (BP) related to the risk of erosive esophagitis (EE) in patients undergoing endoscopy. **(A)** Forest plot, **(B)** funnel plot. CI, confidence interval; IV, inverse variance; SE, standard error.

**A**

**B**

**Supplementary Figure S13.** Odds ratio (OR) of dyslipidemia related to the risk of erosive esophagitis (EE) in patients undergoing endoscopy. **(A)** Forest plot, **(B)** funnel plot. CI, confidence interval; IV, inverse variance; SE, standard error.

**A**

**B**

**Supplementary Figure S14.** Odds ratio (OR) of hypertriglyceridemia ≥150 mg/dL (vs. <150 mg/dL) related to the risk of erosive esophagitis (EE) in patients undergoing endoscopy. **(A)** Forest plot, **(B)** funnel plot. CI, confidence interval; IV, inverse variance; SE, standard error.

**A**

**B**

**Supplementary Figure S15.** Odds ratio (OR) of hypercholesterolemia ≥200 mg/dL (vs. <200 mg/dL) related to the risk of erosive esophagitis (EE) in patients undergoing endoscopy. **(A)** Forest plot, **(B)** funnel plot. CI, confidence interval; IV, inverse variance; SE, standard error.

**A**

**B**

**Supplementary Figure S16.** Odds ratio (OR) of high low density lipoprotein cholesterol (LDL-C) ≥130mg/dL (vs. <130 mg/dL) related to the risk of erosive esophagitis (EE) in patients undergoing endoscopy. **(A)** Forest plot, **(B)** funnel plot. CI, confidence interval; IV, inverse variance; SE, standard error.

**A**

**B**

**Supplementary Figure S17.** Odds ratio (OR) of low high density lipoprotein cholesterol (HDL-C) <40mg/dL (vs. ≥40 mg/dL) for male and <50 mg/dL (vs. ≥50 mg/dL) for female related to the risk of erosive esophagitis (EE) in patients undergoing endoscopy. **(A)** Forest plot, **(B)** funnel plot. CI, confidence interval; IV, inverse variance; SE, standard error.

**A B**

**Supplementary Figure S18.** Odds ratio (OR) of hiatal hernia (HH) related to the risk of erosive esophagitis (EE) in patients undergoing endoscopy. **(A)** Forest plot, **(B)** funnel plot. CI, confidence interval; IV, inverse variance; SE, standard error.

**A**

**B**

**Supplementary Figure S19.** Odds ratio (OR) of *H. pylori* infection related to the risk of erosive esophagitis (EE) in patients undergoing endoscopy. **(A)** Forest plot, **(B)** funnel plot. CI, confidence interval; IV, inverse variance; SE, standard error.

**A**

**B**

**Supplementary Figure S20.** Odds ratio (OR) of gastric ulcer related to the risk of erosive esophagitis (EE) in patients undergoing endoscopy. **(A)** Forest plot, **(B)** funnel plot. CI, confidence interval; IV, inverse variance; SE, standard error.

**A**

**B**

**Supplementary Figure S21.** Odds ratio (OR) of duodenal ulcer related to the risk of erosive esophagitis (EE) in patients undergoing endoscopy. **(A)** Forest plot, **(B)** funnel plot. CI, confidence interval; IV, inverse variance; SE, standard error.

**A**

**B**

**Supplementary Figure S22.** Odds ratio (OR) of atrophic gastritis related to the risk of erosive esophagitis (EE) in patients undergoing endoscopy. **(A)** Forest plot, **(B)** funnel plot. CI, confidence interval; IV, inverse variance; SE, standard error.

**A**

**B**

**Supplementary Figure S23.** Odds ratio (OR) of atrophic gastritis related to the risk of erosive esophagitis (EE) in patients undergoing endoscopy. **(A)** Forest plot, **(B)** funnel plot. CI, confidence interval; IV, inverse variance; SE, standard error.

**A**

**B**

**Supplementary Figure S24.** Odds ratio (OR) of non-steroidal anti-inflammatory drug (NSAID) use related to the risk of erosive esophagitis (EE) in patients undergoing endoscopy. **(A)** Forest plot, **(B)** funnel plot. CI, confidence interval; IV, inverse variance; SE, standard error.

**A**

**B**

**Supplementary Figure S25.** Odds ratio (OR) of aspirin use related to the risk of erosive esophagitis (EE) in patients undergoing endoscopy. **(A)** Forest plot, **(B)** funnel plot. CI, confidence interval; IV, inverse variance; SE, standard error.

**A**

**B**

**Supplementary Figure S26.** Odds ratio (OR) of non-steroidal anti-inflammatory drug (NSAID) and/or aspirin use related to the risk of erosive esophagitis (EE) in patients undergoing endoscopy. **(A)** Forest plot, **(B)** funnel plot. CI, confidence interval; IV, inverse variance; SE, standard error.

**A**

**B**

**Supplementary Figure S27.** Odds ratio (OR) of proton pump inhibitor (PPI) use related to the risk of erosive esophagitis (EE) in patients undergoing endoscopy. **(A)** Forest plot, **(B)** funnel plot. CI, confidence interval; IV, inverse variance; SE, standard error.

**A**

**B**

**Supplementary Figure S28.** Odds ratio (OR) of H2 receptor antagonist (H2RA) use related to the risk of erosive esophagitis (EE) in patients undergoing endoscopy. **(A)** Forest plot, **(B)** funnel plot. CI, confidence interval; IV, inverse variance; SE, standard error.

**A**

**B**

**Supplementary Figure S29.** Odds ratio (OR) of antacids use related to the risk of erosive esophagitis (EE) in patients undergoing endoscopy. **(A)** Forest plot, **(B)** funnel plot. CI, confidence interval; IV, inverse variance; SE, standard error.

**Supplementary Figure S30.** Worldwide and continental prevalence meta-analysis of erosive esophagitis (EE). CI, confidence interval; ES, effect size.
