# Supplementary material for: Risk factors and 26-years worldwide prevalence of endoscopic erosive esophagitis from 1997 to 2022: a meta-analysis
Source: Sci Rep. 2023 Sep 14;13:15249. doi: 10.1038/s41598-023-42636-7 (PMC10502104; doi:10.1038/s41598-023-42636-7)
Supplement: Supplementary file 3 — Supplementary Tables. [file 41598_2023_42636_MOESM3_ESM.docx]

**Supplementary Table S1.** Quality assessment of the included case-control studies based on Newcastle-Ottawa Scale (NOS).

| Author | Selection | | | | Comparability of cases and controls on the basis of the design or analysis | Exposure | | | Study Quality | |
| --- | --- | --- | --- | --- | --- | --- | --- | --- | --- | --- |
|  | **Adequateness of the case definition** | **Representativeness of the cases** | **Selection of controls** | **Definition of controls** |  | **Ascertainment of exposure** | **Same method of ascertainment for cases and controls** | **Non-response rate** | **Total score** | **Judgment** |
| Abraham et al.^1^ | * | 0 | 0 | 0 | ** | * | * | * | 6 | Moderate |
| Adekanle et al.^2^ | * | * | 0 | * | ** | * | * | * | 8 | Good |
| Avidan et al.^3^ | * | * | 0 | * | ** | * | * | * | 8 | Good |
| Barreda Costa et al.^4^ | * | * | 0 | * | ** | * | * | * | 8 | Good |
| Chang et al.^5^ | * | * | 0 | * | ** | * | * | * | 8 | Good |
| Cheng et al.^6^ | * | * | 0 | * | ** | * | * | * | 8 | Good |
| Chua et al.^7^ | * | * | 0 | * | ** | * | * | * | 8 | Good |
| Chung SJ et al.^8^ | * | * | 0 | * | ** | * | * | * | 8 | Good |
| El-Serag et al.^9^ | 0 | * | 0 | 0 | ** | * | * | * | 6 | Moderate |
| Filiberti et al.^10^ | * | * | 0 | * | ** | * | * | * | 8 | Good |
| Ham et al.^11^ | * | 0 | 0 | * | * | * | * | * | 6 | Moderate |
| Jones et al.^12^ | * | * | * | 0 | * | * | * | * | 7 | Good |
| Jones et al.^13^ | * | * | 0 | * | ** | * | * | * | 8 | Good |
| Kainuma et al.^14^ | * | 0 | 0 | 0 | ** | * | * | * | 6 | Moderate |
| Kim HY^15^ | * | * | 0 | 0 | ** | * | * | * | 7 | Good |
| Kim JY et al.^16^ | * | * | 0 | * | ** | * | * | * | 8 | Good |
| Lee D. et al.^17^ | * | * | 0 | * | ** | * | * | * | 8 | Good |
| Lee HL et al.^18^ | * | * | 0 | 0 | ** | * | * | * | 7 | Good |
| Lee HL et al.^19^ | * | * | 0 | * | ** | * | * | * | 8 | Good |
| Lee SW et al.^20^ | * | * | 0 | * | ** | * | * | * | 8 | Good |
| Lee SW et al.^21^ | * | * | 0 | * | ** | * | * | * | 8 | Good |
| Li et al.^22^ | * | * | 0 | * | ** | * | * | * | 8 | Good |
| Lippmann et al.^23^ | * | * | 0 | * | ** | * | * | * | 8 | Good |
| Loke et al.^24^ | * | 0 | 0 | 0 | ** | * | * | * | 6 | Moderate |
| Mahdi et al.^25^ | 0 | 0 | 0 | 0 | * | * | * | * | 4 | Moderate |
| Matsuki et al.^26^ | * | * | 0 | * | ** | * | * | * | 8 | Good |
| Oikawa et al.^27^ | * | 0 | 0 | * | * | * | * | * | 6 | Moderate |
| Park JH et al.^28^ | * | * | 0 | * | ** | * | * | * | 8 | Good |
| Park CH et al.^29^ | * | * | 0 | 0 | ** | * | * | * | 7 | Good |
| Rafat et al.^30^ | * | * | 0 | * | ** | * | * | * | 8 | Good |
| Sadiku et al.^31^ | * | * | * | 0 | ** | * | * | * | 8 | Good |
| Savarino et al.^32^ | * | * | 0 | * | ** | * | * | * | 8 | Good |
| Shimatani et al.^33^ | 0 | 0 | 0 | * | ** | * | 0 | * | 5 | Moderate |
| Wu et al.^34^ | * | * | 0 | * | ** | * | * | * | 8 | Good |
| Wu et al.^35^ | * | * | 0 | * | ** | * | * | * | 8 | Good |
| Yamamoto et al.^36^ | * | 0 | 0 | 0 | * | * | * | * | 5 | Moderate |

**Supplementary Table S2.** Quality assessment of the included cohort studies based on Newcastle-Ottawa Scale (NOS).

| Author | Selection | | | | Comparability of cohorts on the basis of the design or analysis | Exposure | | | Study Quality | |
| --- | --- | --- | --- | --- | --- | --- | --- | --- | --- | --- |
|  | **Representativeness of the exposed cohort** | **Selection of the non-exposed cohort** | **Ascertainment of exposure** | **Demonstration that outcome of interest was not present at start of study** |  | **Assessment of outcome** | **Enough follow-up time length for outcome to occur** | **Adequacy of follow-up of cohorts** | **Total score** | **Judgment** |
| Chen et al.^37^ | 0 | * | * | * | ** | * | * | * | 8 | Good |
| Cheng et al.^38^ | * | * | * | 0 | ** | * | 0 | 0 | 6 | Moderate |
| Chue et al.^39^ | 0 | * | * | * | ** | * | * | 0 | 7 | Good |
| Chung H et al.^40^ | * | * | * | * | ** | * | * | * | 9 | Good |
| Chung TH et al.^41^ | * | * | * | * | ** | * | * | * | 9 | Good |
| Hung HH et al.^42^ | * | * | * | * | * | * | 0 | 0 | 6 | Moderate |
| Isshi et al.^43^ | 0 | * | * | * | ** | * | * | * | 8 | Good |
| Jonaitis L et al.^44^ | 0 | * | * | * | ** | * | * | * | 8 | Good |
| Kavitt et al.^45^ | 0 | * | * | * | ** | * | 0 | 0 | 6 | Moderate |
| Kim SY et al.^46^ | * | * | * | * | ** | * | 0 | 0 | 7 | Good |
| Koo et al.^47^ | * | * | * | * | ** | * | * | * | 9 | Good |
| Kulig et al.^48^ | * | * | * | * | ** | * | * | * | 9 | Good |
| Lee ES et al.^49^ | 0 | * | * | * | ** | * | * | * | 8 | Good |
| Lee H et al.^50^ | * | * | * | * | ** | * | * | * | 9 | Good |
| Matsumura et al.^51^ | 0 | * | * | 0 | * | * | 0 | 0 | 5 | Moderate |
| Migaczewski et al.^52^ | 0 | * | * | * | ** | * | * | * | 8 | Good |
| Yang et al.^53^ | * | * | * | * | ** | * | * | * | 9 | Good |

**Supplementary Table S3.** Quality assessment of the included cross-sectional studies based on Newcastle-Ottawa Scale (NOS).

| Author | Selection | | | | Comparability of subjects in different outcome groups on the basis of design or analysis | Exposure | | Study Quality | |
| --- | --- | --- | --- | --- | --- | --- | --- | --- | --- |
|  | **Representativeness of the sample** | **Sample size** | **Non-respondents** | **Ascertainment of exposure** |  | **Assessment of outcome** | **Statistical test** | **Total score** | **Judgment** |
| Al Shammaa et al.^54^ | 0 | * | * | ** | ** | ** | * | 9 | Very good |
| Avidan et al.^55^ | 0 | 0 | * | ** | ** | ** | * | 8 | Good |
| Avidan et al.^56^ | * | * | * | * | ** | ** | * | 9 | Very good |
| Baeg et al.^57^ | * | * | * | ** | ** | ** | * | 10 | Very good |
| Chiba et al.^58^ | * | * | * | * | ** | ** | * | 9 | Very good |
| Chih et al.^59^ | * | * | * | * | ** | ** | * | 9 | Very good |
| Cho et al.^60^ | * | * | * | ** | ** | ** | * | 10 | Very good |
| Choi et al.^61^ | 0 | 0 | * | ** | * | ** | * | 8 | Good |
| Chung TH et al.^62^ | 0 | * | * | * | ** | ** | * | 8 | Good |
| Deppe et al.^63^ | 0 | 0 | 0 | ** | ** | ** | * | 7 | Good |
| El-Serag et al.^64^ | 0 | 0 | 0 | * | ** | ** | * | 6 | Satisfactory |
| El-Serag et al.^65^ | * | * | * | ** | ** | ** | * | 10 | Very good |
| Fujiwara et al.^66^ | * | 0 | 0 | ** | ** | ** | * | 8 | Good |
| Gaddam et al.^67^ | * | * | * | * | ** | ** | * | 9 | Very good |
| Gado et al.^68^ | * | * | * | ** | ** | ** | * | 10 | Very good |
| Gatoupulou et al.^69^ | 0 | 0 | * | * | * | ** | * | 6 | Satisfactory |
| Gunji et al.^70^ | 0 | * | * | ** | ** | ** | * | 9 | Very good |
| Ha et al.^71^ | * | 0 | * | ** | ** | ** | * | 9 | Very good |
| Heo et al.^72^ | * | * | * | * | ** | ** | * | 9 | Very good |
| Hsieh et al.^73^ | * | * | * | * | ** | ** | * | 9 | Very good |
| Hsu et al.^74^ | 0 | 0 | * | * | ** | ** | * | 7 | Good |
| Hung WC et al.^75^ | * | * | * | ** | ** | ** | * | 10 | Very good |
| Hung WC et al.^76^ | * | * | * | * | ** | ** | * | 9 | Very good |
| Jo et al.^77^ | * | * | * | * | ** | ** | * | 9 | Very good |
| Jonaitis LV et al.^78^ | 0 | 0 | * | * | ** | ** | * | 7 | Good |
| Jung et al.^79^ | 0 | 0 | * | * | ** | ** | * | 7 | Good |
| Kang et al.^80^ | * | * | * | * | ** | ** | * | 9 | Very good |
| Kawai et al.^81^ | * | * | * | ** | ** | ** | * | 10 | Very good |
| Kim JG et al.^82^ | 0 | * | * | * | ** | ** | * | 8 | Good |
| Ko et al.^83^ | 0 | * | * | ** | ** | ** | * | 9 | Very good |
| Lee YC et al.^84^ | * | * | * | * | ** | ** | * | 9 | Very good |
| Lee SD et al.^85^ | 0 | 0 | * | * | * | ** | * | 6 | Satisfactory |
| Lee SW et al.^86^ | 0 | 0 | * | * | * | ** | * | 6 | Satisfactory |
| Lee SW et al.^87^ | * | * | * | * | ** | ** | * | 9 | Very good |
| Lien et al.^88^ | * | * | * | * | ** | ** | * | 9 | Very good |
| Lord et al.^89^ | 0 | 0 | * | * | ** | ** | * | 7 | Good |
| Matsuda et al.^90^ | 0 | * | * | * | ** | ** | * | 8 | Good |
| Meira et al.^91^ | * | * | * | * | ** | ** | * | 9 | Very good |
| Minatsuki et al.^92^ | * | * | * | * | ** | ** | * | 9 | Very good |
| Mun et al.^93^ | 0 | * | * | * | ** | ** | * | 8 | Good |
| Nam et al.^94^ | * | * | * | * | ** | ** | * | 9 | Very good |
| Noh et al.^95^ | * | * | 0 | ** | ** | ** | * | 9 | Very good |
| Nurleili et al.^96^ | 0 | 0 | 0 | * | ** | ** | 0 | 5 | Satisfactory |
| Ohashi et al.^97^ | * | * | * | * | ** | ** | * | 9 | Very good |
| Ou et al.^98^ | * | * | * | * | ** | ** | * | 9 | Very good |
| Ronkainen et al.^99^ | * | * | * | * | ** | ** | * | 9 | Very good |
| Savarino et al.^100^ | * | * | 0 | * | ** | ** | * | 8 | Good |
| Shaker et al.^101^ | 0 | * | * | * | ** | ** | * | 9 | Very good |
| Shimamoto et al.^102^ | * | * | * | ** | ** | ** | * | 10 | Very good |
| Sogabe et al.^103^ | 0 | 0 | * | * | ** | ** | * | 7 | Good |
| Sogabe et al.^104^ | * | * | 0 | ** | * | ** | * | 9 | Very good |
| Tai et al.^105^ | 0 | 0 | * | * | ** | ** | * | 7 | Good |
| Tai et al.^106^ | 0 | * | * | * | ** | ** | * | 8 | Good |
| Vaishnav et al.^107^ | * | 0 | * | * | * | ** | * | 7 | Good |
| Wang FW et al.^108^ | * | * | * | ** | ** | ** | * | 10 | Very good |
| Wang PC et al.^109^ | 0 | 0 | * | ** | ** | ** | * | 8 | Good |
| Wang K et al.^110^ | * | * | * | * | ** | ** | * | 9 | Very good |
| Wei et al.^111^ | * | * | * | * | ** | ** | * | 9 | Very good |
| Yasuhara et al.^112^ | * | * | 0 | ** | ** | ** | * | 9 | Very good |
| Ye et al.^113^ | * | * | * | * | ** | ** | * | 9 | Very good |
| Ze et al.^114^ | 0 | * | * | * | ** | ** | * | 8 | Good |

**Supplementary Table References**

1. Abraham, A. *et al.* Erosive Esophagitis in the Obese: The Effect of Ethnicity and Gender on Its Association. *Gastroenterol. Res. Pract.* **2016**, 7897390 (2016).

2. Adekanle, O., Olowookere, S. A., Ijarotimi, O., Ndububa, D. A. & Komolafe, A. O. Relationship between body mass index, waist circumference, waist hip ratio and erosive gastroesophageal reflux disease in a tertiary centre in Nigeria: A case control study. *Alexandria J. Med.* **54**, 605–609 (2018).

3. Avidan, B., Sonnenberg, A., Schnell, T. G. & Sontag, S. J. Risk factors for erosive reflux esophagitis: a case-control study. *Am. J. Gastroenterol.* **96**, 41–46 (2001).

4. Barreda Costa, C. *et al.* [Lower prevalence of Helicobacter pylori infection observed in patients with erosive esophagitis]. *Rev. Gastroenterol. del Peru organo Of. la Soc. Gastroenterol. del Peru* **34**, 33–37 (2014).

5. Chang, C.-H. *et al.* Alcohol and tea consumption are associated with asymptomatic erosive esophagitis in Taiwanese men. *PLoS One* **12**, e0173230 (2017).

6. Cheng, H.-H., Chang, C.-S., Wang, H.-J. & Wang, W.-C. Interleukin-1beta and -10 polymorphisms influence erosive reflux esophagitis and gastritis in Taiwanese patients. *J. Gastroenterol. Hepatol.* **25**, 1443–1451 (2010).

7. Chua, C.-S. *et al.* Metabolic risk factors associated with erosive esophagitis. *J. Gastroenterol. Hepatol.* **24**, 1375–1379 (2009).

8. Chung, S. J. *et al.* Metabolic syndrome and visceral obesity as risk factors for reflux oesophagitis: a cross-sectional case-control study of 7078 Koreans undergoing health check-ups. *Gut* **57**, 1360–1365 (2008).

9. El-Serag, H. B. & Sonnenberg, A. Association of esophagitis and esophageal strictures with diseases treated with nonsteroidal anti-inflammatory drugs. *Am. J. Gastroenterol.* **92**, 52–56 (1997).

10. Filiberti, R. A. *et al.* Alcohol consumption pattern and risk of Barrett’s oesophagus and erosive oesophagitis: an Italian case-control study. *Br. J. Nutr.* **117**, 1151–1161 (2017).

11. Ham, H. *et al.* Esophagogastric junction contractile integral and morphology: Two high-resolution manometry metrics of the anti-reflux barrier. *J. Gastroenterol. Hepatol.* **32**, 1443–1449 (2017).

12. Jones, M. P. *et al.* Hiatal hernia size is the dominant determinant of esophagitis presence and severity in gastroesophageal reflux disease. *Am. J. Gastroenterol.* **96**, 1711–1717 (2001).

13. Jones, M. P., Sloan, S. S., Jovanovic, B. & Kahrilas, P. J. Impaired egress rather than increased access: an important independent predictor of erosive oesophagitis. *Neurogastroenterol. Motil. Off. J. Eur. Gastrointest. Motil. Soc.* **14**, 625–631 (2002).

14. Kainuma, M. *et al.* The association between objective tongue color and endoscopic findings: results from the Kyushu and Okinawa population study (KOPS). *BMC Complement. Altern. Med.* **15**, 372 (2015).

15. Kim, H. Y. Association Between Erosive Esophagitis and the Anthropometric Index in the General Korean Population. *Balkan Med. J.* **36**, 169–173 (2019).

16. Kim, J. Y. *et al.* Association of sleep dysfunction and emotional status with gastroesophageal reflux disease in Korea. *J. Neurogastroenterol. Motil.* **19**, 344–354 (2013).

17. Lee, D., Lee, K. J., Kim, K. M. & Lim, S. K. Prevalence of asymptomatic erosive esophagitis and factors associated with symptom presentation of erosive esophagitis. *Scand. J. Gastroenterol.* **48**, 906–912 (2013).

18. Lee, H. L. *et al.* Association between GERD-related erosive esophagitis and obesity. *J. Clin. Gastroenterol.* **42**, 672–675 (2008).

19. Lee, H. L. *et al.* Association between erosive esophagitis and visceral fat accumulation quantified by abdominal CT scan. *J. Clin. Gastroenterol.* **43**, 240–243 (2009).

20. Lee, S.-W. *et al.* Characteristics of symptom presentation and risk factors in patients with erosive esophagitis and nonerosive reflux disease. *Med. Princ. Pract. Int. J. Kuwait Univ. Heal. Sci. Cent.* **23**, 460–464 (2014).

21. Lee, S.-W. *et al.* Impact of Obesity on a Chinese Population with Erosive Esophagitis and Barrett’s Esophagus. *Gut Liver* **11**, 377–382 (2017).

22. Li, C.-H. *et al.* Different risk factors between reflux symptoms and mucosal injury in gastroesophageal reflux disease. *Kaohsiung J. Med. Sci.* **31**, 320–327 (2015).

23. Lippmann, Q. K., Crockett, S. D., Dellon, E. S. & Shaheen, N. J. Quality of life in GERD and Barrett’s esophagus is related to gender and manifestation of disease. *Am. J. Gastroenterol.* **104**, 2695–2703 (2009).

24. Loke, S.-S., Yang, K. D., Chen, K.-D. & Chen, J.-F. Erosive esophagitis associated with metabolic syndrome, impaired liver function, and dyslipidemia. *World J. Gastroenterol.* **19**, 5883–5888 (2013).

25. Mahdi, B. M., Hasan, R. M. & Salih, W. H. Human leukocyte antigen HLADRB1 determinants susceptibility to gastroesophageal reflux disease. *Arq. Gastroenterol.* **54**, 41–45 (2017).

26. Matsuki, N. *et al.* Lifestyle factors associated with gastroesophageal reflux disease in the Japanese population. *J. Gastroenterol.* **48**, 340–349 (2013).

27. Oikawa, T. *et al.* Gene polymorphisms of NOD1 and interleukin-8 influence the susceptibility to erosive esophagitis in Helicobacter pylori infected Japanese population. *Hum. Immunol.* **73**, 1184–1189 (2012).

28. Park, J.-H. *et al.* Metabolic syndrome is associated with erosive esophagitis. *World J. Gastroenterol.* **14**, 5442–5447 (2008).

29. Park, C. H. *et al.* Differences in the risk factors of reflux esophagitis according to age in Korea. *Dis. esophagus* **27**, 116–121 (2012).

30. Rafat, M. N. *et al.* Adiponectin level changes among Egyptians with gastroesophageal reflux disease. *JGH open an open access J. Gastroenterol. Hepatol.* **2**, 21–27 (2018).

31. Sadiku, E. *et al.* Extra-esophageal symptoms in individuals with and without erosive esophagitis: a case-control study in Albania. *BMC Gastroenterol.* **21**, 76 (2021).

32. Savarino, E. *et al.* Characteristics of reflux episodes and symptom association in patients with erosive esophagitis and nonerosive reflux disease: study using combined impedance-pH off therapy. *Am. J. Gastroenterol.* **105**, 1053–1061 (2010).

33. Shimatani, T. *et al.* Gastric acid normosecretion is not essential in the pathogenesis of mild erosive gastroesophageal reflux disease in relation to Helicobacter pylori status. *Dig. Dis. Sci.* **49**, 787–794 (2004).

34. Wu, P. *et al.* The association of metabolic syndrome with reflux esophagitis: a case-control study. *Neurogastroenterol. Motil. Off. J. Eur. Gastrointest. Motil. Soc.* **23**, 989–994 (2011).

35. Wu, P. *et al.* Dietary intake and risk for reflux esophagitis: a case-control study. *Gastroenterol. Res. Pract.* **2013**, 691026 (2013).

36. Yamamoto, T. *et al.* Prevalence of erosive esophagitis among Japanese patients taking low-dose aspirin. *J. Gastroenterol. Hepatol.* **25**, 792–794 (2010).

37. Chen, C.-N., Wu, M.-S., Lien, G.-S. & Suk, F.-M. Influence of replacing percutaneous endoscopic gastrostomy for nasogastric tube feeding on gastroesophageal reflux disease with erosive esophagitis. *Adv. Dig. Med.* **3**, 49–55 (2016).

38. Cheng, H. *et al.* Supplementation of Los Angeles classification with esophageal mucosa index of hemoglobin can predict the treatment response of erosive reflux esophagitis. *Surg. Endosc.* **25**, 2478–2486 (2011).

39. Chue, K. M. *et al.* The Hill’s Classification Is Useful to Predict the Development of Postoperative Gastroesophageal Reflux Disease and Erosive Esophagitis After Laparoscopic Sleeve Gastrectomy. *J. Gastrointest. Surg. Off. J. Soc. Surg. Aliment. Tract* **26**, 1162–1170 (2022).

40. Chung, H. *et al.* Noninvasive Prediction of Erosive Esophagitis Using a Controlled Attenuation Parameter (CAP)-Based Risk Estimation Model. *Dig. Dis. Sci.* **61**, 507–516 (2016).

41. Chung, T.-H., Lee, J., Jeong, I.-D. & Lee, K.-C. Effect of Weight Changes on the Development of Erosive Esophagitis. *Korean J. Fam. Med.* **41**, 14–19 (2020).

42. Hung, H.-H. *et al.* Establishing a risk scoring system for predicting erosive esophagitis. *Adv. Dig. Med.* **3**, 95–100 (2016).

43. Isshi, K. *et al.* Clinical features and therapeutic responses to proton pump inhibitor in patients with severe reflux esophagitis: A multicenter prospective observational study. *JGH open an open access J. Gastroenterol. Hepatol.* **5**, 99–106 (2021).

44. Jonaitis, L., Kupčinskas, J., Kiudelis, G. & Kupčinskas, L. De novo erosive esophagitis in duodenal ulcer patients related to pre-existing reflux symptoms, smoking, and patient age, but not to Helicobacter pylori eradication: a one-year follow-up study. *Medicina (Kaunas).* **46**, 454–459 (2010).

45. Kavitt, R. T. *et al.* Esophageal Mucosal Impedance Pattern is Distinct in Patients With Extraesophageal Reflux Symptoms and Pathologic Acid Reflux. *J. Voice* **31**, 347–351 (2017).

46. Kim, S. Y. *et al.* Gender Specific Differences in Prevalence and Risk Factors for Gastro-Esophageal Reflux Disease. *J. Korean Med. Sci.* **34**, e158 (2019).

47. Koo, J. S. *et al.* Abdominal obesity as a risk factor for the development of erosive esophagitis in subjects with a normal esophago-gastric junction. *Gut Liver* **3**, 276–284 (2009).

48. Kulig, M. *et al.* Risk factors of gastroesophageal reflux disease: methodology and first epidemiological results of the ProGERD study. *J. Clin. Epidemiol.* **57**, 580–589 (2004).

49. Lee, E. S. *et al.* Comparison of risk factors and clinical responses to proton pump inhibitors in patients with erosive oesophagitis and non-erosive reflux disease. *Aliment. Pharmacol. Ther.* **30**, 154–164 (2009).

50. Lee, H. *et al.* Relationship between obesity and development of erosive reflux disease: A mediation analysis of the role of cardiometabolic risk factors. *Sci. Rep.* **7**, 6375 (2017).

51. Matsumura, T. *et al.* Endoscopic-Guided Measurement of Mucosal Admittance can Discriminate Gastroesophageal Reflux Disease from Functional Heartburn. *Clin. Transl. Gastroenterol.* **8**, e94 (2017).

52. Migaczewski, M. *et al.* The prevalence of, and risk factors for, Barrett’s oesophagus after sleeve gastrectomy. *Wideochirurgia i inne Tech. maloinwazyjne = Videosurgery other miniinvasive Tech.* **16**, 710–714 (2021).

53. Yang, Y.-J., Sheu, B.-S., Chang, W.-L., Cheng, H.-C. & Yang, H.-B. Increased body mass index after H. pylori eradication for duodenal ulcer predisposes to erosive reflux esophagitis. *J. Clin. Gastroenterol.* **43**, 705–710 (2009).

54. Al Shammaa, A. S. M., Abdulmuhsen, F. K. & Hatem, R. M. Fatty liver disease and the risk of erosive esophagitis in a sample of Iraqi patients: A cross sectional study. *Rev. Latinoam. Hipertens.* **15**, 8–14 (2021).

55. Avidan, B., Sonnenberg, A., Schnell, T. G., Budiman-Mak, E. & Sontag, S. J. Risk factors of oesophagitis in arthritic patients. *Eur. J. Gastroenterol. Hepatol.* **13**, 1095–1099 (2001).

56. Avidan, B., Sonnenberg, A., Schnell, T. G. & Sontag, S. J. Acid reflux is a poor predictor for severity of erosive reflux esophagitis. *Dig. Dis. Sci.* **47**, 2565–2573 (2002).

57. Baeg, M. K., Ko, S.-H., Ko, S. Y., Jung, H. S. & Choi, M.-G. Obesity increases the risk of erosive esophagitis but metabolic unhealthiness alone does not: a large-scale cross-sectional study. *BMC Gastroenterol.* **18**, 82 (2018).

58. Chiba, H. *et al.* A cross-sectional study on the risk factors for erosive esophagitis in young adults. *Intern. Med.* **51**, 1293–1299 (2012).

59. Chih, P.-C. *et al.* Overweight associated with increased risk of erosive esophagitis in a non-obese Taiwanese population. *PLoS One* **8**, e77932 (2013).

60. Cho, J. H. *et al.* Old age and male sex are associated with increased risk of asymptomatic erosive esophagitis: analysis of data from local health examinations by the Korean National Health Insurance Corporation. *J. Gastroenterol. Hepatol.* **26**, 1034–1038 (2011).

61. Choi, J. S. *et al.* Fatty liver disease and the risk of erosive oesophagitis in the Korean population: a cross-sectional study. *BMJ Open* **9**, e023585 (2019).

62. Chung, T. H., Lee, J. & Kim, M. C. Impact of night-shift work on the prevalence of erosive esophagitis in shipyard male workers. *Int. Arch. Occup. Environ. Health* **89**, 961–966 (2016).

63. Deppe, H. *et al.* Erosive esophageal reflux vs. non erosive esophageal reflux: oral findings in 71 patients. *BMC Oral Health* **15**, 84 (2015).

64. El-Serag, H. B., Satia, J. A. & Rabeneck, L. Dietary intake and the risk of gastro-oesophageal reflux disease: a cross sectional study in volunteers. *Gut* **54**, 11–17 (2005).

65. El-Serag, H. B., Graham, D. Y., Satia, J. A. & Rabeneck, L. Obesity is an independent risk factor for GERD symptoms and erosive esophagitis. *Am. J. Gastroenterol.* **100**, 1243–1250 (2005).

66. Fujiwara, Y. *et al.* Differences in clinical characteristics between patients with endoscopy-negative reflux disease and erosive esophagitis in Japan. *Am. J. Gastroenterol.* **100**, 754–758 (2005).

67. Gaddam, S. *et al.* The impact of pre-endoscopy proton pump inhibitor use on the classification of non-erosive reflux disease and erosive oesophagitis. *Aliment. Pharmacol. Ther.* **32**, 1266–1274 (2010).

68. Gado, A., Ebeid, B., Abdelmohsen, A. & Axon, A. Prevalence of reflux esophagitis among patients undergoing endoscopy in a secondary referral hospital in Giza, Egypt. *Alexandria J. Med.* **51**, 89–94 (2015).

69. Gatopoulou, A. *et al.* Impact of Helicobacter pylori infection on histological changes in non-erosive reflux disease. *World J. Gastroenterol.* **10**, 1180–1182 (2004).

70. Gunji, T. *et al.* Risk factors for erosive esophagitis: a cross-sectional study of a large number of Japanese males. *J. Gastroenterol.* **46**, 448–455 (2011).

71. Ha, N. R. *et al.* Differences in clinical characteristics between patients with non-erosive reflux disease and erosive esophagitis in Korea. *J. Korean Med. Sci.* **25**, 1318–1322 (2010).

72. Heo, C. M. *et al.* [Impact of Sarcopenia on the Risk of Erosive Esophagitis]. *Korean J. Gastroenterol.* **75**, 132–140 (2020).

73. Hsieh, Y.-H. *et al.* What is the impact of metabolic syndrome and its components on reflux esophagitis? A cross-sectional study. *BMC Gastroenterol.* **19**, 33 (2019).

74. Hsu, C.-S. *et al.* Increasing insulin resistance is associated with increased severity and prevalence of gastro-oesophageal reflux disease. *Aliment. Pharmacol. Ther.* **34**, 994–1004 (2011).

75. Hung, W.-C. *et al.* Nonalcoholic fatty liver disease vs. obesity on the risk of erosive oesophagitis. *Eur. J. Clin. Invest.* **44**, 1143–1149 (2014).

76. Hung, W.-C. *et al.* Gender differences in the association of non-alcoholic fatty liver disease and metabolic syndrome with erosive oesophagitis: a cross-sectional study in a Taiwanese population. *BMJ Open* **6**, e013106 (2016).

77. Jo, S. Y. *et al.* Comparison of gastroesophageal reflux disease symptoms and proton pump inhibitor response using gastroesophageal reflux disease impact scale questionnaire. *J. Neurogastroenterol. Motil.* **19**, 61–69 (2013).

78. Jonaitis, L. V, Kiudelis, G. & Kupcinskas, L. Characteristics of patients with erosive and nonerosive GERD in high-Helicobacter-pylori prevalence region. *Dis. esophagus Off. J. Int. Soc. Dis. Esophagus* **17**, 223–227 (2004).

79. Jung, J. G. *et al.* Vegetarianism as a protective factor for reflux esophagitis: a retrospective, cross-sectional study between Buddhist priests and general population. *Dig. Dis. Sci.* **58**, 2244–2252 (2013).

80. Kang, M. S. *et al.* Abdominal obesity is an independent risk factor for erosive esophagitis in a Korean population. *J. Gastroenterol. Hepatol.* **22**, 1656–1661 (2007).

81. Kawai, T. *et al.* Helicobacter pylori infection and reflux esophagitis in young and middle-aged Japanese subjects. *J. Gastroenterol. Hepatol.* **25 Suppl 1**, S80-5 (2010).

82. Kim, J. G. *et al.* Halimeter ppb Levels as the Predictor of Erosive Gastroesophageal Reflux Disease. *Gut Liver* **4**, 320–325 (2010).

83. Ko, S.-H., Baeg, M. K., Jung, H. S., Kim, P. & Choi, M.-G. Russian Caucasians have a higher risk of erosive reflux disease compared with East Asians: A direct endoscopic comparison. *Neurogastroenterol. Motil. Off. J. Eur. Gastrointest. Motil. Soc.* **29**, (2017).

84. Lee, Y.-C. *et al.* Comparative analysis between psychological and endoscopic profiles in patients with gastroesophageal reflux disease: a prospective study based on screening endoscopy. *J. Gastroenterol. Hepatol.* **21**, 798–804 (2006).

85. Lee, S. D., Keum, B., Chun, H. J. & Bak, Y.-T. Gastroesophageal Reflux Disease in Type II Diabetes Mellitus With or Without Peripheral Neuropathy. *J. Neurogastroenterol. Motil.* **17**, 274–278 (2011).

86. Lee, S.-W. *et al.* Impact of body mass index and gender on quality of life in patients with gastroesophageal reflux disease. *World J. Gastroenterol.* **18**, 5090–5095 (2012).

87. Lee, S.-W. *et al.* Association of metabolic syndrome with erosive esophagitis and Barrett’s esophagus in a Chinese population. *J. Chin. Med. Assoc.* **80**, 15–18 (2017).

88. Lien, H.-C. *et al.* Increasing prevalence of erosive esophagitis among Taiwanese aged 40 years and above: a comparison between two time periods. *J. Clin. Gastroenterol.* **43**, 926–932 (2009).

89. Lord, R. V. N. *et al.* Hiatal hernia, lower esophageal sphincter incompetence, and effectiveness of Nissen fundoplication in the spectrum of gastroesophageal reflux disease. *J. Gastrointest. Surg. Off. J. Soc. Surg. Aliment. Tract* **13**, 602–610 (2009).

90. Matsuda, R. *et al.* Gastroesophageal Reflux Disease-Related Disorders of Systemic Sclerosis Based on the Analysis of 66 Patients. *Digestion* **98**, 201–208 (2018).

91. Meira, A. T. D. S., Tanajura, D. & Viana, I. D. S. CLINICAL AND ENDOSCOPIC EVALUATION IN PATIENTS WITH GASTROESOPHAGEAL SYMPTOMS. *Arq. Gastroenterol.* **56**, 51–54 (2019).

92. Minatsuki, C. *et al.* Background factors of reflux esophagitis and non-erosive reflux disease: a cross-sectional study of 10,837 subjects in Japan. *PLoS One* **8**, e69891 (2013).

93. Mun, E., Kim, D., Lee, Y., Lee, W. & Park, S. Association between Shift Work and Reflux Esophagitis: The Kangbuk Samsung Health Study. *Int. J. Environ. Res. Public Health* **18**, (2021).

94. Nam, S. Y. *et al.* Different effects of dietary factors on reflux esophagitis and non-erosive reflux disease in 11,690 Korean subjects. *J. Gastroenterol.* **52**, 818–829 (2017).

95. Noh, Y. W., Jung, H.-K., Kim, S.-E. & Jung, S.-A. Overlap of Erosive and Non-erosive Reflux Diseases With Functional Gastrointestinal Disorders According to Rome III Criteria. *J. Neurogastroenterol. Motil.* **16**, 148–156 (2010).

96. Nurleili, R. A. *et al.* Visceral fat thickness of erosive and non-erosive reflux disease subjects in Indonesia’s tertiary referral hospital. *Diabetes Metab. Syndr.* **13**, 1929–1933 (2019).

97. Ohashi, S. *et al.* Visceral fat obesity is the key risk factor for the development of reflux erosive esophagitis in 40-69-years subjects. *Esophagus* **18**, 889–899 (2021).

98. Ou, J.-L. *et al.* Prevalence and risk factors of erosive esophagitis in Taiwan. *J. Chin. Med. Assoc.* **75**, 60–64 (2012).

99. Ronkainen, J. *et al.* High prevalence of gastroesophageal reflux symptoms and esophagitis with or without symptoms in the general adult Swedish population: a Kalixanda study report. *Scand. J. Gastroenterol.* **40**, 275–285 (2005).

100. Savarino, E. *et al.* Overweight is a risk factor for both erosive and non-erosive reflux disease. *Dig. liver Dis. Off. J. Ital. Soc. Gastroenterol. Ital. Assoc. Study Liver* **43**, 940–945 (2011).

101. Shaker, A. & Magdy, M. Frequency of obstructive sleep apnea (OSA) in patients with gastroesophageal reflux disease (GERD) and the effect of nasal continuous positive airway pressure. *Egypt. J. Chest Dis. Tuberc.* **65**, 797–803 (2016).

102. Shimamoto, T. *et al.* No association of coffee consumption with gastric ulcer, duodenal ulcer, reflux esophagitis, and non-erosive reflux disease: a cross-sectional study of 8,013 healthy subjects in Japan. *PLoS One* **8**, e65996 (2013).

103. Sogabe, M., Okahisa, T., Kimura, Y., Hibino, S. & Yamanoi, A. Visceral fat predominance is associated with erosive esophagitis in Japanese men with metabolic syndrome. *Eur. J. Gastroenterol. Hepatol.* **24**, 910–916 (2012).

104. Sogabe, M. *et al.* Differences in Several Factors in the Development of Erosive Esophagitis Among Patients at Various Stages of Metabolic Syndrome: A Cross-Sectional Study. *Diabetes. Metab. Syndr. Obes.* **14**, 1589–1600 (2021).

105. Tai, C.-M. *et al.* The relationship between visceral adiposity and the risk of erosive esophagitis in severely obese Chinese patients. *Obesity (Silver Spring).* **18**, 2165–2169 (2010).

106. Tai, C.-M. *et al.* Increase in gastroesophageal reflux disease symptoms and erosive esophagitis 1 year after laparoscopic sleeve gastrectomy among obese adults. *Surg. Endosc.* **27**, 1260–1266 (2013).

107. Vaishnav, B., Bamanikar, A., Maske, P., Reddy, A. & Dasgupta, S. Gastroesophageal Reflux Disease and its Association with Body Mass Index: Clinical and Endoscopic Study. *J. Clin. Diagn. Res.* **11**, OC01–OC04 (2017).

108. Wang, F.-W. *et al.* Erosive esophagitis in asymptomatic subjects: risk factors. *Dig. Dis. Sci.* **55**, 1320–1324 (2010).

109. Wang, P.-C. *et al.* Male sex, hiatus hernia, and Helicobacter pylori infection associated with asymptomatic erosive esophagitis. *J. Gastroenterol. Hepatol.* **27**, 586–591 (2012).

110. Wang, K. *et al.* A population-based survey of gastroesophageal reflux disease in a region with high prevalence of esophageal cancer in China. *Chin. Med. J. (Engl).* **132**, 1516–1523 (2019).

111. Wei, T.-Y., Hsueh, P.-H., Wen, S.-H., Chen, C.-L. & Wang, C.-C. The role of tea and coffee in the development of gastroesophageal reflux disease. *Ci ji yi xue za zhi = Tzu-chi Med. J.* **31**, 169–176 (2019).

112. Yasuhara, H. *et al.* Large waist circumference is a risk factor for reflux esophagitis in Japanese males. *Digestion* **81**, 181–187 (2010).

113. Ye, B.-X. *et al.* Association between body mass index, hiatal hernia and gastroesophageal reflux in patients with gastroesophageal reflux disease. *Shijie Huaren Xiaohua Zazhi* **20**, 3375–3379 (2012).

114. Ze, E. Y., Kim, B. J., Kang, H. & Kim, J. G. Abdominal Visceral to Subcutaneous Adipose Tissue Ratio Is Associated with Increased Risk of Erosive Esophagitis. *Dig. Dis. Sci.* **62**, 1265–1271 (2017).
